# Unsupervised Feature Selection via Self-Supervised HSIC and Elastic Net Regularization

**DOI:** 10.3390/e28091027

**Published:** 2026-09-16

**Authors:** Yuhong Chen, Tinghua Wang, Long Zou

**Affiliations:** Key Laboratory of Data Science and Artificial Intelligence of Jiangxi Education Institutes, Gannan Normal University, Ganzhou 341000, China; 1240740013@gnnu.edu.cn (Y.C.); 1240741007@gnnu.edu.cn (L.Z.)

**Keywords:** unsupervised feature selection, Hilbert–Schmidt independence criterion, centered kernel alignment, elastic net, redundancy penalty, stability

## Abstract

Unsupervised feature selection is essential for high-dimensional data analysis, where irrelevant and redundant variables may obscure the intrinsic data structure, reduce interpretability, and degrade downstream learning performance. The key challenge is to identify informative features without label supervision while suppressing redundant selections under nonlinear dependencies. To address this issue, this paper proposes an unsupervised Hilbert–Schmidt Independence Criterion (HSIC)–Elastic Net (ENet) feature selection framework, termed U-HSIC-ENet. The proposed method reformulates unlabeled feature weighting as a self-supervised kernel alignment problem. Specifically, a target kernel is constructed directly from unlabeled data to encode global sample relationships, while each feature is represented by a centered and Frobenius-normalized feature-induced kernel. Feature relevance is then measured by centered kernel alignment (CKA) in a reproducing kernel Hilbert space (RKHS). The main novelty of U-HSIC-ENet lies in a unified relevance–redundancy–stability formulation. Feature–target alignment is used to estimate nonlinear relevance, whereas pairwise similarities between feature-induced kernels are used to characterize inter-feature redundancy in the same kernel alignment space. On this basis, an explicit off-diagonal redundancy penalty is incorporated into a nonnegative Elastic Net-type objective, which strengthens the suppression of co-selected similar features while preserving sparse and stable feature weighting. The resulting quadratic formulation clarifies how relevance promotion, redundancy control, sparsity, and numerical stabilization are coupled within a single optimization framework. Experiments on eight benchmark datasets under a fixed-budget evaluation protocol show that U-HSIC-ENet achieves the strongest average performance on Normalized Mutual Information (NMI), the Adjusted Rand Index (ARI), and clustering accuracy (ACC) compared with representative graph-, spectral-, and HSIC-based baselines. The advantage is the most pronounced on NMI, suggesting that the self-supervised target kernel and CKA-based relevance modeling are effective in preserving the clustering-relevant nonlinear structure. Friedman tests and Wilcoxon signed-rank tests with Holm correction provide statistical support for the observed improvements. Subsampling-based stability evaluation reveals a trade-off between clustering effectiveness and selection reproducibility: several baselines achieve higher stability scores despite the stronger average clustering performance of U-HSIC-ENet. These results indicate that the proposed framework is effective for unsupervised nonlinear feature weighting when relevance estimation, redundancy control, and stability are considered jointly.

## 1. Introduction

High-dimensional data are common in modern data analysis and machine learning applications, where the number of measured variables can be large relative to the number of samples. Many variables may be irrelevant, noisy, or redundant, which can obscure the intrinsic sample structure, reduce model interpretability, and degrade downstream learning performance. Feature selection addresses this problem by selecting a compact subset of informative variables and has become an important component of efficient and interpretable learning pipelines [1]. From a data-centered perspective, feature selection is also closely related to redundancy reduction, structure preservation, and model generalization in high-dimensional learning [2]. In the unsupervised setting, the problem becomes more difficult because no class labels are available to define feature relevance directly. Instead, the structural information used for selection must be inferred from the unlabeled data distribution itself [3].

A major class of unsupervised feature selection methods derives proxy supervision from the geometry of unlabeled data, most commonly through graph- or spectral-based formulations. The Laplacian Score evaluates each feature according to its locality-preserving ability on a nearest-neighbor graph [4]. SPEC further connects feature ranking with spectral graph analysis and evaluates features according to their consistency with the spectral structure of the data [5]. MCFS uses spectral embeddings and sparse regression to identify features that preserve the multi-cluster structure [6]. More recently, robust unsupervised feature selection based on fuzzy anchor graphs has shown that adaptive structural modeling remains important for characterizing cluster relationships in unlabeled data [7].

From an optimization perspective, graph- and spectral-based methods usually encode data structure through neighborhood graphs, Laplacian smoothness, or sparse regression on spectral embeddings. Although these methods are effective in many scenarios, they may be sensitive to graph construction, neighborhood size, and affinity parameters. Moreover, their structural criteria are mainly based on geometric smoothness or graph-preserving ability, whereas they do not directly quantify nonlinear statistical dependence between individual features and the latent structure of unlabeled data.

Kernel dependence measures provide a different route for modeling nonlinear relationships. The Hilbert–Schmidt Independence Criterion (HSIC) measures statistical dependence through the Hilbert–Schmidt norm of a cross-covariance operator, and its empirical form can be written using traces of centered kernel matrices [8]. A recent review of HSIC-based learning also shows that the HSIC is useful as a model-free dependence criterion in a wide range of learning tasks [9]. This kernel matrix viewpoint is attractive for feature selection because a target-side structural signal can be represented as a kernel matrix rather than as explicit class labels. HSIC Lasso is the most direct dependence-based reference for the present work, since it formulates feature selection as a feature-wise kernelized sparse learning problem [10]. Related studies have incorporated the HSIC into multiple-kernel learning [11], feature-weighted fuzzy support vector machines [12], and broader HSIC Lasso-based feature selection analysis [13]. These works indicate that kernel dependence is a powerful tool for nonlinear feature evaluation, but they do not by themselves resolve the unsupervised target construction and redundancy control problems considered in this paper.

Another important issue is the relevance–redundancy trade-off. In high-dimensional data, several features may be strongly related to the same underlying structure but highly similar to each other. Selecting many such features can lead to redundant subsets and weaker interpretability. Mutual information-based feature selection first emphasized the need to consider both informativeness and redundancy [14]. Yu and Liu further analyzed relevance and redundancy in efficient feature selection [15]. The mRMR criterion formalized the max-relevance and min-redundancy principle for selecting compact feature subsets [16]. Brown later provided a unifying information-theoretic interpretation of several feature selection criteria [17]. These studies motivate relevance–redundancy modeling, but they mainly rely on information-theoretic formulations rather than normalized kernel alignment representations.

Sparse regularization is also central to feature selection. The $l_{1}$-norm is widely used to induce sparse solutions by shrinking uninformative feature weights to zero. However, purely $l_{1}$-based selection may be unstable when features are strongly correlated, because it often retains only one arbitrary representative from a correlated feature group. Elastic Net regularization addresses this limitation by combining the sparsity-inducing effect of the $l_{1}$-norm with the stabilization effect of the $l_{2}$-norm [18]. Stability selection further stresses that selected feature subsets should remain reliable under data perturbations [19], and later work on feature selection stability provides formal criteria for evaluating reproducibility [20]. These results suggest that sparsity alone is not sufficient in correlated high-dimensional settings; stable feature weighting should also be considered.

Centered kernel alignment (CKA) provides a useful way to connect nonlinear dependence measurement with scale-comparable kernel similarity. Cortes et al. developed centered alignment as a normalized criterion for comparing kernels [21]. Wang et al. reviewed kernel alignment and its applications, showing that alignment-based criteria can measure structural similarity between kernels [22]. Kornblith et al. popularized CKA for comparing neural network representations, which further illustrates the interpretability of normalized kernel similarity [23]. CKA-inspired learning has also been studied in fuzzy support vector machines [24]. In this paper, CKA is not treated as a dependence measure independent of the HSIC. Instead, it is used as a normalized form of the centered HSIC trace term so that feature–target relevance and feature–feature redundancy can be evaluated on a common scale.

Despite these advances, existing unsupervised feature selection methods still leave a modeling gap. Graph- and spectral-based methods are effective for preserving local or spectral structure, but their criteria are mainly based on graph smoothness or embedding consistency rather than direct nonlinear dependence between individual features and the latent structure of unlabeled data. HSIC Lasso provides a stronger dependence-driven perspective, but its standard formulation mainly emphasizes feature–target relevance and sparse weighting. Redundancy among selected feature-induced kernels is only implicitly controlled by the quadratic interaction term. Meanwhile, sparse selection can be unstable when correlated features carry similar structural information, which motivates the use of Elastic Net-type stabilization. Therefore, a desirable unsupervised feature selection model should jointly address four questions: how to construct a target-side structural signal without labels, how to measure nonlinear feature relevance in a scale-comparable manner, how to suppress redundant co-selection explicitly, and how to maintain stable sparse feature weighting.

Motivated by these considerations, this paper proposes an unsupervised HSIC–Elastic Net (ENet) feature selection framework, termed U-HSIC-ENet. Rather than simply combining the HSIC with Elastic Net regularization, U-HSIC-ENet reformulates unsupervised feature weighting as a self-supervised relevance–redundancy–stability modeling problem. In the absence of class labels, a target-side kernel is constructed directly from the data matrix to encode global sample relationships. Each feature is then represented by a centered and Frobenius-normalized feature-induced kernel, and its relevance is quantified by CKA with respect to the self-supervised target kernel. Meanwhile, pairwise similarities between feature-induced kernels are used to characterize inter-feature redundancy, and an explicit off-diagonal redundancy penalty is introduced to discourage the co-selection of structurally similar features. Together with Elastic Net-type sparse stabilization, this formulation provides a unified framework for nonlinear relevance estimation, redundancy suppression, and stable feature weighting in the unsupervised setting.

The proposed framework is evaluated under a fixed-budget feature selection protocol and compared with representative graph-, spectral-, and dependence-based baselines. HSIC Lasso is included as the most relevant dependence-based baseline because it uses feature-wise kernels and sparse nonnegative weights for feature selection [10]. Multi-Cluster Unsupervised Nonlinear Feature Selection (MUNFS) is also considered as a recent manifold-assisted nonlinear baseline; it combines Uniform Manifold Approximation and Projection (UMAP)-based manifold representation [25] with multi-cluster nonlinear unsupervised feature selection based on joint manifold learning and generalized Lasso [26]. In addition, unsupervised nonlinear feature selection on signed networks shows that nonlinear dependence modeling remains useful in high-dimensional unlabeled settings [27]. Recent work on distribution alignment-based unsupervised feature selection further emphasizes the joint role of global distribution matching and local structure preservation [28]. A unified view of HSIC-based feature selection also supports the relevance of dependence measures for feature selection [29]. Manifold regularization provides the classical geometric background for exploiting unlabeled data structure [30]. More recent studies have explored self-supervised graph auto-encoders for feature selection [31] and similarity graph-guided unsupervised feature selection for multiview clustering [32]. Kernel-based dependence research has also continued to extend the HSIC to more general statistical dependence testing settings [33]. Together, these studies indicate that structural learning and kernel dependence modeling remain active directions for extracting informative representations from unlabeled data.

Beyond unsupervised feature selection, the broader high-dimensional variable selection literature also emphasizes the reliability and reproducibility of regularized selection. In cancer genomics, Fan et al. [34] reviewed frequentist and Bayesian high-dimensional inference and highlighted that variable selection results should be accompanied by appropriate uncertainty assessment and reproducibility considerations, particularly when sparse regularization is applied to complex high-dimensional data. From a Bayesian perspective, shrinkage priors provide a complementary mechanism for controlling high-dimensional coefficients. Fan et al. [35] investigated the horseshoe, horseshoe+, and regularized horseshoe priors for robust high-dimensional regression, showing that global–local shrinkage can support variable selection and uncertainty quantification even without enforcing exact sparsity. These perspectives complement the present Elastic Net-type formulation: whereas Bayesian shrinkage focuses on posterior regularization and uncertainty quantification, U-HSIC-ENet focuses on stable unsupervised feature weighting through sparse regularization, $l_{2}$-type stabilization, and explicit redundancy control.

The main contributions of this work are summarized as follows. First, we propose U-HSIC-ENet, an unsupervised HSIC-ENet feature selection framework that reformulates unlabeled feature weighting as a self-supervised kernel alignment problem. By constructing a target kernel directly from unlabeled data, the proposed method provides a data-driven structural signal for nonlinear feature relevance estimation without requiring external labels. Second, we establish a unified kernel alignment representation for both feature relevance and feature redundancy. Feature relevance is measured by the CKA between each feature-induced kernel and the self-supervised target kernel, whereas redundancy is characterized by pairwise similarities among feature-induced kernels. This formulation makes the relevance–redundancy trade-off explicit in an RKHS. Third, we introduce an explicit off-diagonal redundancy penalty into a nonnegative Elastic Net-type objective. Unlike the implicit redundancy effect contained in the original alignment-induced quadratic term, the proposed penalty directly strengthens the suppression of co-selected similar features, thereby improving subset diversity while retaining sparse feature weighting and an $l_{2}$-based stabilization mechanism. Fourth, we derive an equivalent quadratic formulation and solve the resulting composite objective by proximal gradient optimization. This derivation clarifies how relevance promotion, redundancy suppression, sparsity, and $l_{2}$-type stabilization are coupled in a single optimization model. Finally, extensive experiments on eight benchmark datasets are conducted under a fixed-budget protocol. The results, together with Friedman tests, Wilcoxon signed-rank tests with Holm correction, win/tie/loss analysis, subsampling stability evaluation, and budget robustness analysis, show that U-HSIC-ENet provides a favorable balance between clustering effectiveness and selection robustness. The remainder of this paper is organized as follows. Section 2 introduces the preliminaries. Section 3 presents the proposed formulation and optimization procedure. Section 4 reports the experimental setup and results. Section 5 concludes this paper.

## 2. Preliminaries

### 2.1. Notation

Let $X=[x_{1},\ldots ,x_{n}{]}^{⊤}\in R^{n\times d}$ denote a dataset with n samples and d features, where $x_{i}\in R^{d}$ is the i-th sample. The k-th feature vector is denoted by $x^{\left(k\right)}\in R^{n}$, corresponding to the k-th column of X. Throughout this paper, bold upright uppercase letters denote matrices, bold italic lowercase letters denote vectors, and ordinary italic letters denote scalars. The Frobenius inner product between two matrices A and B of the same size is defined as $\left\langle A,B\rangle_{F}=tr(A^{⊤}B\right)$, and the corresponding Frobenius norm is $\|A{\|}_{F}=\sqrt{\langle A,A\rangle_{F}}$. To center kernel matrices, we use the centering matrix

(1) $$H=I_{n}-\frac{1}{n}11^{⊤},$$

where $I_{n}$ is the identity matrix, and 1 is the all-ones vector. For any generic kernel matrix $K\in R^{n\times n}$, its centered version is denoted by

(2) $$\bar{K}=HKH.$$

This notation follows the common centered kernel alignment convention: an original kernel matrix and its centered version are distinguished by an overbar. This convention will be applied to both the self-supervised target kernel and the feature-wise kernels in the following subsections.

### 2.2. Hilbert–Schmidt Independence Criterion

A central issue in feature selection is how to measure the statistical dependence between variables. In kernel methods, the Hilbert–Schmidt Independence Criterion (HSIC) provides a principled dependence measure by comparing kernel embeddings in reproducing kernel Hilbert spaces (RKHSs) [8]. Let K and L be kernel matrices constructed from two variables of interest. The empirical estimator of the HSIC is given by

(3) $$HSIC(K,L)=\frac{1}{(n-1{)}^{2}}tr(KHLH),$$

which evaluates the degree of dependence between two variables through their kernel representations. A larger value indicates stronger statistical dependence.

Since the constant factor does not affect optimization with respect to feature weights, the essential part of the HSIC reduces to the centered trace term $tr(KHLH)=tr(HKHHLH)=\langle \bar{K},\bar{L}\rangle_{F}$. This formulation reveals that the HSIC can be interpreted as the Frobenius inner product between centered kernel matrices. Such a representation is particularly suitable for feature selection, because it connects dependence measurement with kernel alignment: if the kernel induced by a feature is well aligned with a target-side kernel, then the feature is structurally informative. This idea forms the basis of HSIC Lasso and subsequent kernel-based feature selection frameworks [10].

### 2.3. Centered Kernel Alignment and Its Relation to HSIC

Kernel Target Alignment (KTA) measures the similarity between two kernels through the normalized Frobenius inner product of their kernel matrices. Given two kernel matrices $K_{1}$ and $K_{2}$, KTA is defined as

(4) $$KTA(K_{1},K_{2})=\frac{\langle K_{1},K_{2}\rangle_{F}}{\sqrt{\langle K_{1},K_{1}\rangle_{F}\langle K_{2},K_{2}\rangle_{F}}}$$

Centered kernel alignment (CKA) improves KTA by first centering the kernel matrices. Given two original kernel matrices $K_{1}$ and $K_{2}$, their centered versions are ${\bar{K}}_{1}=HK_{1}H,{\bar{K}}_{2}=HK_{2}H$. The CKA between $K_{1}$ and $K_{2}$ is then defined as

(5) $$\begin{array}{cc} CKA(K_{1},K_{2}) & =\frac{\langle {\bar{K}}_{1},{\bar{K}}_{2}\rangle_{F}}{\sqrt{\langle {\bar{K}}_{1},{\bar{K}}_{1}\rangle_{F}\langle {\bar{K}}_{2},{\bar{K}}_{2}\rangle_{F}}}\\ & =\frac{\langle {\bar{K}}_{1},{\bar{K}}_{2}\rangle_{F}}{\|{\bar{K}}_{1}{\|}_{F}\|{\bar{K}}_{2}{\|}_{F}}. \end{array}$$

Compared with KTA, the essential difference in CKA lies in the centering operation. Centering reduces the influence of feature space origin shifts and improves the reliability of kernel similarity evaluation. Moreover, because the numerator $\langle {\bar{K}}_{1},{\bar{K}}_{2}\rangle_{F}$ corresponds to the centered trace term in the HSIC, CKA can be viewed as a normalized version of the HSIC. Therefore, CKA retains the nonlinear dependence measurement ability of the HSIC while providing a scale-comparable similarity score between kernel matrices [21].

### 2.4. Self-Supervised Target Kernel Construction

In supervised HSIC-based feature selection, the target kernel is constructed from class labels. In the unsupervised setting, however, no label information is available, and the structural signal must be derived directly from the data. To address this issue, we adopt a self-supervised target kernel construction strategy, where the dataset itself serves as a proxy source of structural supervision. This strategy is motivated by HSIC-based feature selection, where the target-side information can be represented through a kernel matrix [10], and is also related to recent self-supervised or structure learning-based feature selection studies that construct structural signals from unlabeled data [31,32].

Specifically, a Gaussian radial basis function (RBF) kernel is defined on the sample space as

(6) $$L_{ij}=exp\left(-\frac{\|x_{i}-x_{j}{\|}_{2}^{2}}{2\sigma^{2}}\right),i,j=1,\ldots ,n,$$

where σ >0 is the kernel bandwidth parameter of the self-supervised target kernel. Following the general centering rule in Equation (2), the centered self-supervised target kernel is obtained as

(7) $$\bar{L}=HLH.$$

Here, L is the original self-supervised target kernel matrix, whereas $\bar{L}$ denotes its centered version. This construction uses global pairwise sample relationships as a proxy target-side structural signal. Intuitively, if the centered kernel induced by a feature is highly aligned with $\bar{L}$, then the corresponding feature preserves the intrinsic sample relationship encoded by the entire dataset.

The full-data target provides a common structural reference for evaluating all features. However, because the feature being evaluated also contributes to the construction of this target, its relevance score may be affected by self-inclusion. The score should therefore be interpreted as alignment with the observed full-data structure, rather than evidence of relevance to an independently constructed target. Centering and Frobenius normalization do not remove the evaluated feature from the target construction.

### 2.5. Feature-Wise Kernels, Relevance, and Redundancy

To evaluate individual features, a kernel is constructed for each feature separately. For the k-th feature vector $x^{\left(k\right)}$, a one-dimensional Gaussian kernel is defined as

(8) $$K_{ij}^{\left(k\right)}=exp\left(-\frac{{\left(x_{i}^{\left(k\right)}-x_{j}^{\left(k\right)}\right)}^{2}}{2\sigma_{k}^{2}}\right),i,j=1,\ldots ,n,$$

where $\sigma_{k}>0$ is the bandwidth parameter for the k-th feature. The centered feature-wise kernel matrix is

(9) $${\bar{K}}^{\left(k\right)}=HK^{\left(k\right)}H,$$

Here, $K^{\left(k\right)}$ denotes the original kernel matrix induced by the k-th feature, while ${\bar{K}}^{\left(k\right)}$ denotes its centered version. Based on the CKA definition, the relevance of the k-th feature is defined as the CKA between its feature-induced kernel and the self-supervised target kernel:

(10) $$\begin{array}{cc} b_{k}\; & =CKA\left(K^{\left(k\right)},L\right)\\ & =\frac{\langle {\bar{K}}^{\left(k\right)},\bar{L}\rangle_{F}}{\sqrt{\langle {\bar{K}}^{\left(k\right)},{\bar{K}}^{\left(k\right)}\rangle_{F}\langle \bar{L},\bar{L}\rangle_{F}}}\\ & =\frac{\langle {\bar{K}}^{\left(k\right)},\bar{L}\rangle_{F}}{\|{\bar{K}}^{\left(k\right)}{\|}_{F}\|\bar{L}{\|}_{F}}. \end{array}$$

A larger $b_{k}$ indicates that the k-th feature induces a sample similarity structure more consistent with the self-supervised target structure. Therefore, $b_{k}$ quantifies nonlinear feature relevance in the centered kernel alignment space. Similarly, redundancy between the i-th and j-th features is defined as the CKA between their feature-induced kernels:

(11) $$\begin{array}{cc} Q_{ij}\; & =CKA\left(K^{\left(i\right)},K^{\left(j\right)}\right)\\ & =\frac{\langle {\bar{K}}^{\left(i\right)},{\bar{K}}^{\left(j\right)}\rangle_{F}}{\sqrt{\langle {\bar{K}}^{\left(i\right)},{\bar{K}}^{\left(i\right)}\rangle_{F}\langle {\bar{K}}^{\left(j\right)},{\bar{K}}^{\left(j\right)}\rangle_{F}}}\\ & =\frac{\langle {\bar{K}}^{\left(i\right)},{\bar{K}}^{\left(j\right)}\rangle_{F}}{\|{\bar{K}}^{\left(i\right)}{\|}_{F}\|{\bar{K}}^{\left(j\right)}{\|}_{F}}. \end{array}$$

A larger $Q_{ij}$ indicates that two features induce more similar sample relationship structures and are therefore more redundant. In this way, feature relevance and feature redundancy are modeled in the same CKA-based space, and the vector b measures feature–target alignment, while the matrix Q measures pairwise feature–kernel alignment. This formulation follows the relevance–redundancy principle of feature selection [16] and the normalized kernel alignment view of CKA [21].

### 2.6. Elastic Net Regularization

Sparse regularization is widely used to obtain compact feature subsets. The $l_{1}$-norm promotes sparsity by shrinking irrelevant feature weights to zero, but purely $l_{1}$-based sparse selection may become unstable when features are strongly correlated. Elastic Net regularization combines the sparsity-inducing effect of the $l_{1}$-norm with the stabilization effect of the $l_{2}$-norm and is therefore suitable for correlated high-dimensional variables [18].

In the proposed framework, the $l_{1}$ term is used to induce sparse nonnegative feature weights, while the $l_{2}$ term is incorporated into the quadratic interaction matrix to improve numerical conditioning and stabilize feature weighting. Therefore, Elastic Net regularization provides a natural mechanism for balancing sparsity and stability in CKA-based unsupervised feature weighting.

## 3. Proposed Method: U-HSIC-ENet

### 3.1. Theoretical Derivation from Kernel Alignment

In the unsupervised setting, the central question is how to evaluate the usefulness of each feature without access to class labels. Following dependence-based feature selection, informative features are expected to induce sample relationship structures that are highly consistent with the latent structural relationships of the data [8]. HSIC Lasso provides a useful kernelized sparse learning view, in which feature-wise kernel matrices are linearly combined to approximate a target-side kernel matrix under an $l_{1}$-regularized nonnegative weighting scheme [10]. Motivated by this formulation, we reformulate unsupervised feature weighting as a CKA-based self-supervised kernel alignment problem. Unlike supervised HSIC Lasso, the target kernel in the proposed method is not constructed from class labels but directly from the unlabeled data itself.

Let $\bar{L}$ and ${\bar{K}}^{\left(k\right)}$ denote the centered self-supervised target kernel and the centered kernel induced by the k-th feature, respectively, as defined in Equations (7) and (9). According to the CKA definition, kernel alignment is computed after centering and Frobenius normalization, which makes kernel similarities scale-comparable [21]. Therefore, we define the weighted composite kernel as

(12) $$K_{w}=\sum_{k=1}^{d}w_{k}\frac{{\bar{K}}^{\left(k\right)}}{\|{\bar{K}}^{\left(k\right)}{\|}_{F}}$$

where $w=[w_{1},\ldots ,w_{d}{]}^{⊤}\in R_{+}^{d}$ is a nonnegative feature weight vector. The objective is to make the weighted combination of normalized centered feature-wise kernels align with the normalized centered self-supervised target kernel. Thus, we consider the following CKA-based kernel matching problem:

(13) $$\underset{w\geq 0}{\min}\frac{1}{2}{\left\|\sum_{k=1}^{d}w_{k}\frac{{\bar{K}}^{\left(k\right)}}{\|{\bar{K}}^{\left(k\right)}{\|}_{F}}-\frac{\bar{L}}{\|\bar{L}{\|}_{F}}\right\|}_{F}^{2}$$

This formulation follows the Frobenius loss structure of HSIC Lasso [10] while replacing the supervised output kernel with a self-supervised target kernel. The use of centered and Frobenius-normalized kernels follows the normalized centered kernel alignment view of CKA [21]. Substituting the definition of $K_{w}$, the squared Frobenius term can be expanded as

(14) $$\begin{array}{cc} & \frac{1}{2}{\left\|\sum_{k=1}^{d}w_{k}\frac{{\bar{K}}^{\left(k\right)}}{\|{\bar{K}}^{\left(k\right)}{\|}_{F}}-\frac{\bar{L}}{\|\bar{L}{\|}_{F}}\right\|}_{F}^{2}\\ & =\frac{1}{2}\sum_{k=1}^{d}\sum_{l=1}^{d}w_{k}w_{l}Q_{kl}-\sum_{k=1}^{d}w_{k}b_{k}+\frac{1}{2}\\ & =\frac{1}{2}w^{⊤}Qw-b^{⊤}w+\frac{1}{2}. \end{array}$$

According to the CKA definition [21], $b_{k}=\frac{\langle {\bar{K}}^{\left(k\right)},\bar{L}\rangle_{F}}{\|{\bar{K}}^{\left(k\right)}{\|}_{F}\|\bar{L}{\|}_{F}}$ is the CKA score between the k-th feature-induced kernel and the self-supervised target kernel, whereas $Q_{kl}=\frac{{\left\langle {\bar{K}}^{\left(k\right)},{\bar{K}}^{\left(l\right)}\right\rangle }_{F}}{\|{\bar{K}}^{\left(k\right)}{\|}_{F}\|{\bar{K}}^{\left(l\right)}{\|}_{F}}$ is the CKA score between the k-th and l-th feature-induced kernels. Therefore, $b_{k}$ quantifies nonlinear feature relevance with respect to the intrinsic data structure, while $Q_{kl}$ quantifies pairwise structural similarity between two features. Moreover,

(15) $${\left\|\frac{\bar{L}}{\|\bar{L}{\|}_{F}}\right\|}_{F}^{2}=1$$

Because the normalized target kernel has a unit Frobenius norm, the last term in Equation (14) is a constant independent of w, and it can be omitted for optimization. Therefore, Equation (14) leads to the following basic CKA-induced feature weighting objective: $\underset{w\geq 0}{\min}\frac{1}{2}w^{⊤}Qw-b^{⊤}w$.

This formulation reveals a clear relevance–redundancy trade-off. The vector b promotes features that are strongly aligned with the self-supervised target structure, whereas the matrix Q measures pairwise feature–kernel alignment and therefore characterizes redundancy among features. A larger $Q_{kl}$ indicates that two features induce more similar sample relationship structures and are therefore more redundant. In this way, feature relevance and feature redundancy are modeled in the same CKA-based space: b measures feature–target alignment, while Q measures pairwise feature–kernel alignment. This design combines the relevance–redundancy principle of feature selection [16] with the feature-wise kernel matching idea of HSIC Lasso [10] and the normalized kernel alignment view of CKA [21]. Moreover, the positive semidefinite property of Q can be shown directly. Let

(16) $$a_{k}=vec\left(\frac{{\bar{K}}^{\left(k\right)}}{\|{\bar{K}}^{\left(k\right)}{\|}_{F}}\right)\in R^{n^{2}},A=[a_{1},\ldots ,a_{d}]\in R^{n^{2}\times d}$$

(17) $$Q_{kl}=a_{k}^{⊤}a_{l},Q=A^{⊤}A≽0.$$

Therefore, the baseline CKA-induced quadratic term is convex.

### 3.2. Explicit Redundancy Penalization and Regularization

Although the quadratic term induced by Q already contains an implicit redundancy effect, it does not explicitly distinguish diagonal self-contributions from off-diagonal inter-feature redundancy. In practice, redundancy mainly arises from the co-selection of distinct but highly similar features, which is encoded by the off-diagonal entries of Q. To suppress such co-selection more explicitly, we further amplify the off-diagonal part of the interaction matrix. Let $Q_{off}=Q-diag(Q)$, where diag(Q) denotes the diagonal matrix formed by the diagonal entries of Q. Based on this off-diagonal redundancy matrix, the proposed U-HSIC-ENet objective is formulated as

(18) $$\begin{array}{rr} w^{*}= & \arg\min_{w}\frac{1}{2}w^{⊤}Qw-b^{⊤}w+\frac{\gamma }{2}w^{⊤}Q_{off}w+\lambda_{1}\|w{\|}_{1}+\frac{\lambda_{2}}{2}\|w{\|}_{2}^{2},\\ & \;\;s.t.w\geq 0. \end{array}$$

where γ ≥ 0 is the redundancy control parameter, $\lambda_{1}\;\geq \;0$ controls sparsity, and $\lambda_{2}\;\geq \;0$ is the $l_{2}$-stabilization parameter. The term $\frac{\gamma }{2}w^{⊤}Q_{\mathrm{off}}w$ explicitly penalizes the co-selection of features with high pairwise CKA similarity, while $\lambda_{1}\|w{\|}_{1}$ promotes sparse feature weighting. The term $\frac{\lambda_{2}}{2}\|w{\|}_{2}^{2}$ provides the stabilization effect of Elastic Net regularization and improves numerical conditioning under correlated features [18]. We define the modified interaction matrix as

(19) $$M=Q+\gamma Q_{off}+\lambda_{2}I=Q+\gamma \left(Q-diag(Q)\right)+\lambda_{2}I$$

Then the above objective can be equivalently written in the compact quadratic form $\underset{w\geq 0}{\min}\frac{1}{2}w^{⊤}Mw-b^{⊤}w+\lambda_{1}\|w{\|}_{1}$. In this formulation, the linear term ${-b}^{⊤}w$ promotes features that are highly aligned with the intrinsic data structure, whereas the quadratic term $\frac{1}{2}w^{⊤}Mw$ integrates the original feature–kernel interaction, explicit off-diagonal redundancy penalization, and $l_{2}$-type stabilization. Therefore, relevance promotion, redundancy suppression, sparsity, and numerical stabilization are coupled within a unified nonnegative Elastic Net-type kernel alignment objective.

To clarify the relationship with HSIC Lasso, which is the closest methodological reference, we present the original HSIC Lasso objective [10], as summarized in [13]. alongside the U-HSIC-ENet objective in Equation (18), collectively shown in Equation (20), followed by a comparison of their main components.

(20) $$\begin{array}{lllll} & HSICLasso & & U-HSIC-ENet & \\ & \begin{array}{cc} \underset{w\geq 0}{\min}\; & \frac{1}{2}{\left\|{\bar{L}}_{Y}-\sum_{k=1}^{d}w_{k}{\bar{K}}^{\left(k\right)}\right\|}_{F}^{2}\\ & +\lambda \|w{\|}_{1} \end{array} & & \begin{array}{cc} \underset{w\geq 0}{\min}\; & \frac{1}{2}w^{⊤}Qw-b^{⊤}w\\ & +\frac{\gamma }{2}w^{⊤}Q_{off}w\\ & +\lambda_{1}\|w{\|}_{1}+\frac{\lambda_{2}}{2}\|w{\|}_{2}^{2} \end{array} & \end{array}$$

Both formulations use nonnegative sparse feature weights to match a target kernel through feature-wise kernel representations. In the standard HSIC Lasso objective, the target is constructed from supervised responses, whereas U-HSIC-ENet constructs a common target from unlabeled input data. U-HSIC-ENet further uses centered and Frobenius-normalized kernels so that feature relevance and pairwise redundancy are expressed through CKA. Importantly, HSIC Lasso already incorporates implicit redundancy control through its quadratic feature–kernel interactions. The additional term involving $\gamma Q_{off}$ provides separate control over the co-selection of distinct but similar features. Finally, both objectives retain $l_{1}$-regularization for sparsity, while U-HSIC-ENet adds an $l_{2}$-regularization term for numerical stabilization. The proposed formulation therefore combines a data-derived structural target, normalized kernel matching, explicit redundancy control, and Elastic Net-type regularization within a unified objective.

**Proposition** **1.** *Assume that each nondegenerate centered feature-induced kernel is Frobenius-normalized. Then the diagonal entries of the interaction matrix satisfy* $Q_{\mathrm{kk}}=1$ *for all* k *, and consequently* diag(Q)=I *. Therefore, the modified interaction matrix in Equation (19) can be rewritten as*

$$M=(1+\gamma )Q+(\lambda_{2}-\gamma )I$$

*Since* Q ⪰ 0 *according to Equation (17), the minimum eigenvalue of* M *is*

$$\lambda_{\min}(M)=(1+\gamma )\lambda_{\min}(Q)+\lambda_{2}-\gamma$$

 *Hence,* M *is positive semidefinite if and only if*

$$(1+\gamma )\lambda_{\min}(Q)+\lambda_{2}-\gamma \geq 0$$

*A simple data-independent sufficient condition is therefore*

$$\lambda_{2}\geq \gamma .$$

*Under this condition,* M *⪰ 0, and the objective in Equation (18) is convex. Furthermore, if*

$$\lambda_{2}>\gamma ,$$

*then* M *≻ 0, so the objective is strictly convex and admits a unique minimizer. When* $\lambda_{2}<\gamma$*, positive semidefiniteness is not guaranteed and requires*

$$\lambda_{\min}(Q)\geq \frac{\gamma -\lambda_{2}}{1+\gamma }.$$

*Therefore, the convexity and global optimality guarantees of U-HSIC-ENet apply only when the above positive semidefiniteness condition is satisfied.*

**Remark** **1.** *The positive semidefiniteness of* M *is required for convexity, but it is not required for the optimization problem in Equation (18) to be well posed over the nonnegative feasible region. Since each centered feature-induced kernel is positive semidefinite, the Frobenius inner products defining the interaction matrix satisfy* $Q_{kl}\geq 0$ *. Under Frobenius normalization,* $Q_{kk}=1$ *, and therefore*

$$M_{kk}=1+\lambda_{2},\;M_{kl}=(1+\gamma )Q_{kl}\geq 0,\;k\neq l.$$

*Consequently, for any* w≥0 *,*

$$w^{T}Mw=(1+\lambda_{2})\|w{\|}_{2}^{2}+2(1+\gamma )\sum_{k<l}Q_{kl}w_{k}w_{l}\geq (1+\lambda_{2})\|w{\|}_{2}^{2}.$$

*Hence, the objective in Equation (18) is coercive on the nonnegative feasible set and therefore admits at least one global minimizer even when* M *is indefinite. In this case, however, the optimization problem is nonconvex, and the global optimality guarantee for the solution produced by the iterative solver no longer applies.*

### 3.3. Optimization via Proximal Gradient Descent

The optimization properties of Equation (18) depend on the definiteness of the modified interaction matrix M. When the positive semidefiniteness condition in Proposition 1 is satisfied, the objective is a convex composite optimization problem consisting of a smooth quadratic term and a nonsmooth sparse regularizer under nonnegativity constraints. When M is indefinite, the objective is generally nonconvex; nevertheless, as established in Remark 1, it remains coercive and well posed over the nonnegative feasible set. We employ proximal gradient descent in both cases. The convex case admits the standard global convergence guarantee, whereas in the nonconvex case, the iterative procedure is used to seek an approximate stationary feature weight solution without claiming global optimality. We decompose the objective as

(21) $$\begin{array}{rr} F(w) & =f(w)+g(w),\\ \left\{\begin{array}{cc} f(w) & =\frac{1}{2}w^{⊤}Mw-b^{⊤}w,\\ g(w) & =\lambda_{1}\|w{\|}_{1}+\iota_{R_{+}^{d}}(w). \end{array}\right. & \end{array}$$

where $\iota_{R_{+}^{d}}(w)$ denotes the indicator function of the nonnegative orthant. Since the l2-type stabilization has already been absorbed into the quadratic matrix M, the smooth part of the model is completely characterized by f(w), whose gradient is given by

(22) ∇f(w)=Mw−b.

Because f(w) is quadratic, its gradient is Lipschitz continuous with Lipschitz constant $L_{f}=\|M{\|}_{2}$, where $\|M{\|}_{2}$ denotes the spectral norm of M. Accordingly, proximal gradient descent can be implemented with a step size $\eta \in (0,1/L_{f}]$. At iteration t, one first performs a gradient step on the smooth part and then applies the proximal mapping associated with g, as follows. Since M is symmetric and entrywise nonnegative, its spectral norm is determined by its largest eigenvalue. Accordingly, the implementation uses the adaptive step size $\eta =0.9/\lambda_{\max}(M)$, which provides a conservative step size below $1/L_{f}.$

(23) $$v^{\left(t+1\right)}=w^{\left(t\right)}-\eta \left(Mw^{\left(t\right)}-b\right)\;w^{\left(t+1\right)}={prox}_{\eta g}\left(v^{\left(t+1\right)}\right)$$

Because g combines the $l_{1}$-norm and the nonnegativity constraint, the proximal operator reduces to an element-wise positive soft-thresholding mapping. Hence, the update admits the explicit form.

(24) $$\begin{array}{cc} w^{\left(t+1\right)} & =\max\left\{0,v^{\left(t+1\right)}-\eta \lambda_{1}1\right\}\\ & =\max\left\{0,w^{\left(t\right)}-\eta \left(Mw^{\left(t\right)}-b\right)-\eta \lambda_{1}1\right\} \end{array}$$

where the maximum is taken element-wise. This update simultaneously enforces sparsity and nonnegativity while preserving the quadratic interaction structure induced by M. The iteration is repeated until convergence; in implementation, convergence is declared when the relative change between two successive iterates satisfies Equation (24) or when a predefined maximum number of iterations is reached.

(25) $$\frac{\|w^{\left(t+1\right)}-w^{\left(t\right)}{\|}_{2}}{\max\left(1,\|w^{\left(t\right)}{\|}_{2}\right)}<\varepsilon$$

From an optimization viewpoint, the procedure alternates between a gradient step driven by feature relevance and redundancy interactions and a proximal shrinkage step that promotes sparse nonnegative feature weights. When M⪰0, the objective is convex, and proximal gradient descent with a step size satisfying the Lipschitz condition converges to a global minimizer under the standard assumptions of convex composite optimization [36,37]. If M≻0, the objective is strictly convex, and the minimizer is unique. When M is indefinite, the objective becomes nonconvex but remains coercive over the nonnegative feasible set according to Remark 1. In this regime, the same iterative scheme is used to seek an approximate stationary feature weight solution, while no claim of global optimality is made. Based on the final objective and the above optimization strategy, the complete U-HSIC-ENet procedure is summarized in Algorithm 1.
**Algorithm 1. U-HSIC-ENet Optimization Procedure****Input:** data matrix X; sparsity parameter $\lambda_{1}$; stabilization parameter $\lambda_{2}$; redundancy parameter γ; tolerance ε; maximum number of iterations $T_{\max}$ **Output:** learned nonnegative feature-weight vector $\hat{w}$ 1: Construct the self-supervised target kernel L according to Equation (6), and center it as $\bar{L}=HLH$. 2: For each feature *j* = 1, …, *d*, construct $K^{\left(j\right)}$ according to Equation (8), and center it as ${\bar{K}}^{\left(j\right)}=HK^{\left(j\right)}H$. 3: Compute the relevance vector b, where $b_{j}=\frac{\langle {\bar{K}}^{\left(j\right)},\bar{L}\rangle_{F}}{\|{\bar{K}}^{\left(j\right)}{\|}_{F}\|\bar{L}{\|}_{F}}$. 4: Compute the interaction matrix Q, where $Q_{ij}=\frac{\langle {\bar{K}}^{\left(i\right)},{\bar{K}}^{\left(j\right)}\rangle_{F}}{\|{\bar{K}}^{\left(i\right)}{\|}_{F}\|{\bar{K}}^{\left(j\right)}{\|}_{F}}$ 5: Compute the off-diagonal redundancy matrix $Q_{off}=Q-diag(Q)$ 6: Form the modified interaction matrix $M=Q+\gamma Q_{off}+\lambda_{2}I$ 7: Set the step size $\eta =0.9/\lambda_{\max}(M)$. 8: Initialize $w^{\left(0\right)}\in R_{+}^{d}$ (e.g., $w^{\left(0\right)}=0$) and set *t* = 0. 9: Repeat $v^{\left(t+1\right)}=w^{\left(t\right)}-\eta \left(Mw^{\left(t\right)}-b\right)$ 10: Update $w^{\left(t+1\right)}=\max\left\{0,v^{\left(t+1\right)}-\eta \lambda_{1}1\right\}$ 11: t=t+1 12: Until
$$\frac{\|w^{\left(t\right)}-w^{\left(t-1\right)}{\|}_{2}}{\max\left(1,\|w^{\left(t-1\right)}{\|}_{2}\right)}<\varepsilon$$ or $t\geq T_{\max}$ 13: Return $\hat{w}=w^{\left(t\right)}$

When M⪰0, the objective is convex, and the global convergence guarantee applies; $\lambda_{2}\geq \gamma$ is a simple data-independent sufficient condition for this case. When M is indefinite, the objective remains coercive over the nonnegative feasible set according to Remark 1, but the returned solution $\hat{w}$ should be interpreted as a stationary numerical solution rather than a globally optimal one.

### 3.4. Computational Complexity

The computational burden of the proposed method mainly arises from self-supervised target kernel construction, feature-wise kernel construction, CKA-based interaction modeling, and iterative optimization. In a straightforward implementation, constructing the self-supervised target kernel L requires $O(n^{2}d)$, because pairwise sample similarities are computed in the original d-dimensional space. Centering the target kernel to obtain $\bar{L}=\mathrm{HLH}$ requires $O(n^{2})$. Constructing all feature-wise kernels $\{K^{\left(j\right)}{\}}_{j=1}^{d}$ and centering them as ${\bar{K}}^{\left(j\right)}=HK^{\left(j\right)}H$ requires $O(dn^{2})$. Computing the CKA-based relevance vector b requires $O(dn^{2})$, because each centered feature-wise kernel must be compared with the centered target kernel through a normalized Frobenius inner product. The dominant cost arises from constructing the interaction matrix Q, which requires $O(d^{2}n^{2})$, since every pair of centered feature-wise kernels must be compared through CKA-based similarity. This complexity pattern is consistent with the feature-wise kernel construction and kernel interaction mechanism underlying HSIC Lasso-style methods and also explains why computational efficiency and memory reduction have become important issues in scalable HSIC-based learning [10,38,39,40].

During optimization, each proximal gradient iteration is dominated by the matrix–vector multiplication Mw, which costs $O(d^{2})$. If the algorithm runs for T iterations, the corresponding optimization cost is $O(Td^{2})$. Therefore, the total computational complexity of the proposed method is dominated by the construction of Q, especially when the feature dimension d is large.

In terms of memory, storing all feature-wise centered kernels requires $O(dn^{2})$ space, whereas storing Q, $Q_{\mathrm{off}}$, and M requires an additional $O(d^{2})$ space. Therefore, the principal memory bottleneck lies in feature-wise kernel storage in high-dimensional settings. Overall, the proposed method is computationally feasible for moderate-scale datasets, whereas further scalability improvements may rely on block computation, low-rank kernel approximation, Nyström approximation, or compressed interaction modeling, which is consistent with efficiency-oriented developments in the HSIC Lasso literature [38,39,40].

## 4. Experiments

In this section, we evaluate the proposed U-HSIC-ENet on eight benchmark datasets under a fixed-budget unsupervised feature selection protocol. The experimental design is intended to examine not only whether the proposed method improves downstream clustering performance but also whether the proposed relevance–redundancy–stability formulation provides statistically supported evidence over representative graph-based, spectral, and dependence-based baselines. We first describe the datasets, comparison methods, and evaluation measures. We then report fixed-budget clustering performance, followed by statistical significance analysis and subsampling-based stability evaluation. Finally, we examine budget robustness to assess whether the observed advantage persists beyond a single recommended budget.

### 4.1. Comparison Approaches and Performance Measures

We evaluate unsupervised feature selection on eight benchmark datasets, namely Arrhythmia, Wine, Urban Land, Sonar, Dermatology, Parkinsons, Semeion, and Libras. Following the fixed-budget protocol, the number of selected features is set to $k^{*}$, defined as the budget closest to 0.1d, where d denotes the original feature dimensionality. For each dataset, every method produces either a feature ranking or a nonnegative weight vector, and the top-$k^{*}$ features are then retained for downstream clustering evaluation. The dataset statistics and the corresponding fixed budgets are summarized in Table 1. Before feature selection, missing values were imputed using the feature-wise median, zero-variance features were removed, and all retained features were standardized to zero mean and unit variance. Unless otherwise specified, the clustering results were averaged over three independent runs with random seeds 0, 1, and 2 and are reported as the mean ± standard deviation; the best result in each row is highlighted in bold.

We compare U-HSIC-ENet with representative graph-based, spectral, and dependence-based baselines. The Laplacian Score (LS) is used as a locality-preserving graph-based baseline because it ranks features according to their ability to preserve neighborhood relationships [4]. Spectral Feature Selection (SPEC) represents spectral graph-based feature selection and evaluates features through their consistency with the spectral structure of the data [5]. Multi-Cluster Feature Selection (MCFS) uses spectral embeddings and sparse regression to preserve multi-cluster structure [6]. U-HSIC-Lasso is included as a direct dependence-based sparse feature weighting baseline derived from the feature-wise kernelized sparse learning idea of HSIC Lasso [10]. MUNFS-core is used as a manifold-assisted nonlinear baseline; it combines UMAP-based manifold representation [25] with multi-cluster nonlinear unsupervised feature selection based on joint manifold learning and generalized Lasso [26]. This comparison set covers locality-preserving ranking, spectral embedding-based selection, sparse multi-cluster selection, direct HSIC-based weighting, and manifold-assisted nonlinear dependence modeling.

For all Gaussian RBF kernels used in U-HSIC-ENet, the bandwidths were determined automatically using the median heuristic rather than treated as additional tuning parameters. Specifically, the bandwidth of the self-supervised target kernel was set to the median of the positive pairwise Euclidean distances between samples in the standardized full feature space. For each one-dimensional feature-induced kernel, an individual bandwidth was analogously determined from the pairwise distances of that feature. This data-adaptive rule was applied consistently to all datasets and was kept fixed during the subsequent regularization parameter search.

For U-HSIC-ENet, the regularization parameters were selected separately for each dataset from the predefined grids $\lambda_{1}\in \{0.01,0.05,0.1,0.2\}$, $\lambda_{2}\in \{0,0.001,0.01,0.05,0.1\}$, and γ∈{0,0.1,0.3,0.5,1.0}. For benchmark-level hyperparameter selection, each parameter combination was evaluated over all predefined feature budgets and repeated clustering runs, and the combination yielding the highest mean NMI was retained for the final benchmark. Therefore, reference labels were involved in this external benchmark-level hyperparameter selection through NMI, whereas the feature weight learning procedure for any fixed parameter combination itself remains unsupervised and does not use class labels. The selected parameter values for all eight datasets are reported in Table 1.

The proximal gradient solver used a convergence tolerance of $\varepsilon =10^{-7}$ and a maximum of 800 iterations, with the step size adaptively set to $\eta =0.9/\lambda_{\max}(M)$. For the competing methods, fixed predefined implementation settings were used consistently across datasets rather than dataset-specific exhaustive parameter tuning. The LS used five nearest neighbors; U-HSIC-Lasso used $\lambda_{1}=0.1$; and MUNFS-core used $\lambda_{1}=0.1$, a 10-dimensional UMAP representation, 15 neighbors, and a minimum distance of 0.1. For the spectral implementations used in this study, SPEC and MCFS employed a 10-nearest-neighbor spectral embedding, and MCFS used a Lasso coefficient of $10^{-3}$. For SPEC and MCFS, the embedding dimension was set to min{max(2,c+2),max[2,min(10,n−2)]}, where *c* denotes the prescribed number of clusters, and *n* is the number of samples.

After feature selection, K-means clustering was performed in the reduced feature space using the prescribed number of clusters for each benchmark dataset. K-means was initialized with $n_{init}=20$, and the random seed was varied across the three repeated runs. Clustering quality was assessed using Normalized Mutual Information (NMI) [41], the Adjusted Rand Index (ARI) [42], and clustering accuracy (ACC), which provide complementary views of partition consistency and label agreement. NMI measures the information shared between the predicted and reference partitions, whereas the ARI evaluates partition agreement while correcting for chance. ACC is computed as the proportion of correctly assigned samples after optimal cluster–label matching using the Hungarian algorithm [43]. To assess the reliability of the observed differences across multiple datasets and methods, the Friedman test was used as the global nonparametric comparison procedure [44]. Pairwise post hoc comparison follows the multiple-comparison framework discussed by García and Herrera [45], and Holm correction was applied to control the family-wise error rate in pairwise testing [46]. Selection stability is treated as a complementary evaluation criterion following the stability analysis framework of Nogueira et al. [20]. Specifically, the Jaccard index measures selected subset overlap [47], the Kuncheva index adjusts subset overlap by chance agreement under a fixed selection budget [48], and Spearman-based correlation measures the consistency of feature rankings or learned weights [49].

### 4.2. Fixed-Budget Clustering Performance

Table 2, Table 3 and Table 4 report the fixed-budget clustering results in terms of NMI, the ARI, and ACC, respectively. Overall, U-HSIC-ENet achieves the best average performance on all three metrics, with mean NMI =0.4564, mean ARI =0.3272, and mean ACC =0.5895. These results indicate that the proposed dependence- and redundancy-aware weighting framework yields the strongest overall representation among the compared methods under a unified feature budget. The advantage is the most pronounced for NMI.

At the dataset level, U-HSIC-ENet shows the clearest and most consistent advantage on NMI, where it ranks first on all eight datasets. For the ARI, it achieves the best overall average and obtains the highest result on six of the eight datasets. For ACC, it also attains the highest average performance, while several competing methods show strong results on specific datasets. These results indicate that U-HSIC-ENet provides the most favorable overall clustering-oriented performance under the fixed-budget protocol, with particularly consistent advantages in preserving clustering-relevant information measured by NMI.

These results directly support the main modeling motivation of U-HSIC-ENet. The consistent advantage on NMI suggests that the self-supervised target kernel and CKA-based relevance criterion are effective in preserving the clustering-relevant nonlinear structure. The improvement over U-HSIC-Lasso further indicates that Elastic Net-type stabilization and explicit off-diagonal redundancy control provide additional benefits beyond sparse HSIC-based relevance modeling alone. The overall best average performance across NMI, the ARI, and ACC further suggests that the proposed relevance–redundancy–stability formulation improves the quality of selected features under the fixed-budget protocol.

Another noteworthy observation is that the strongest competitors are typically U-HSIC-Lasso and MUNFS-core rather than the purely graph-based or spectral baselines. This pattern is consistent with the design motivation of the proposed method: when nonlinear dependence and feature redundancy jointly matter, HSIC-based selection provides a more informative structural signal than criteria based only on locality preservation or spectral smoothness. Relative to U-HSIC-Lasso, the proposed model further shows that Elastic Net-style stabilization and explicit redundancy control can yield a clear improvement in average clustering quality, especially for NMI and the ARI. Overall, the eight-dataset benchmark provides substantially stronger empirical support for U-HSIC-ENet under the fixed-budget evaluation protocol.

**Figure 1:**
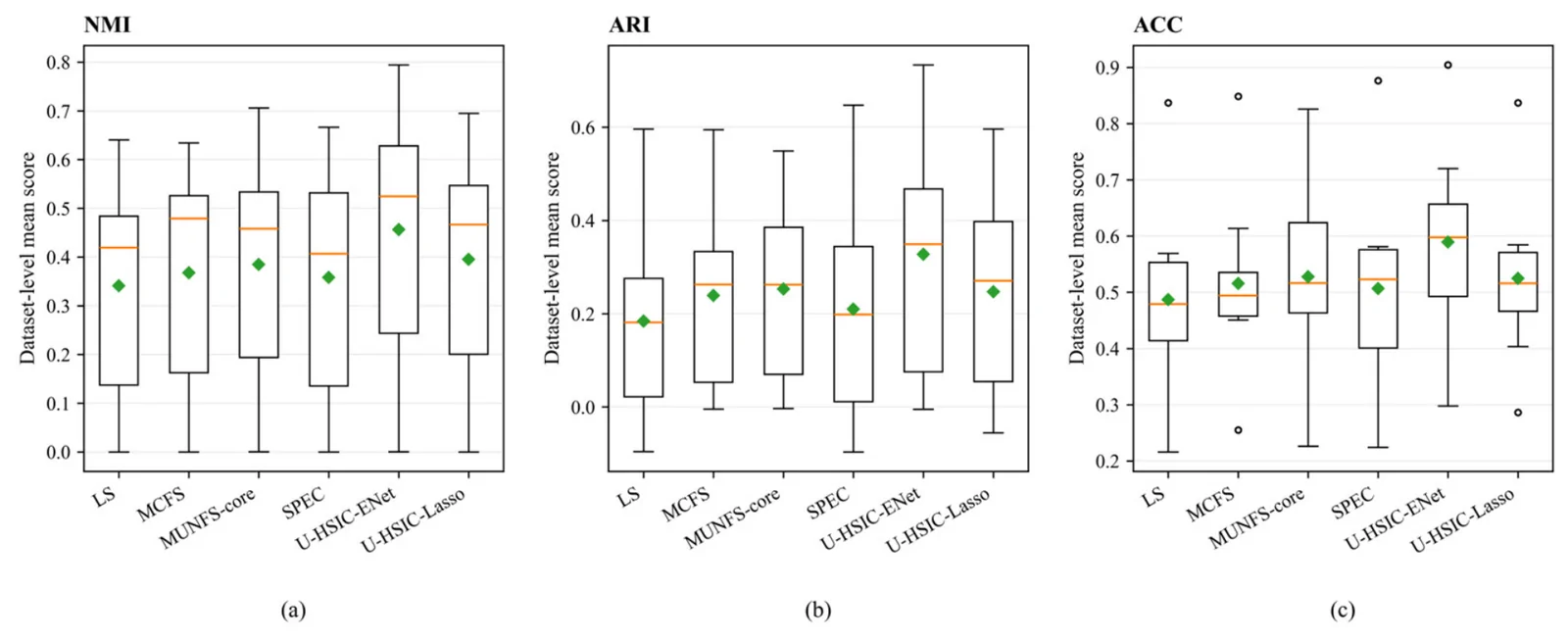
Cross-dataset performance distributions at the fixed budget k*. (**a**) NMI, (**b**) ARI, and (**c**) ACC. Each box summarizes the dataset-level mean scores across the eight datasets. Boxes span the first and third quartiles; orange horizontal lines indicate medians, and green diamonds indicate means. Whiskers extend to the most extreme observations within 1.5 times the interquartile range from the box edges, and open circles indicate observations beyond the whiskers.

To visualize the dataset-level behavior at the recommended budget $k^{*}$, Figure 1 presents the cross-dataset performance distributions of all methods for NMI, the ARI, and ACC. Compared with the row-wise inspection of Table 2, Table 3 and Table 4, the boxplots provide a more direct view of median performance, inter-dataset spread, and distributional robustness. The figure is consistent with the tabulated results in showing that U-HSIC-ENet occupies the most favorable overall position, particularly on NMI, while also maintaining strong average performance on the ARI and ACC.

### 4.3. Statistical Significance Tests

To determine whether the observed performance differences are statistically meaningful across datasets, we further report Friedman tests, pairwise Wilcoxon signed-rank tests with Holm correction, and win/tie/loss summaries. The Friedman test provides a rank-based nonparametric procedure for global comparison across multiple methods and datasets [44]. After the global comparison, pairwise post hoc testing follows the multiple-comparison setting discussed by García and Herrera [45]. Holm correction is then used to adjust pairwise p-values and reduce the risk of false positives caused by repeated testing [46].

As shown in Table 5, the Friedman test is significant for all three metrics under the eight-dataset setting, with p-values below 0.05 for NMI, the ARI, and ACC. Therefore, the null hypothesis that all six methods perform equivalently across datasets can be rejected for every evaluation metric considered here. These results provide global statistical evidence that the performance differences observed in Table 2, Table 3 and Table 4 are not merely descriptive.

The win/tie/loss results in Table 6 show that the advantage of U-HSIC-ENet is broad-based rather than driven by a few favorable datasets. For NMI, U-HSIC-ENet achieves an 8-0-0 record against every baseline, which is fully consistent with its row-wise dominance in Table 2. For the ARI, it records 7-0-1 against the Laplacian Score, MCFS, SPEC, and U-HSIC-Lasso and 6-0-2 against MUNFS-core. For ACC, it remains competitive, with 7-0-1 against the Laplacian Score and U-HSIC-Lasso, 6-0-2 against MCFS and MUNFS-core, and 7-1-0 against SPEC. These counts indicate that the observed gains are systematic at the dataset level rather than consequences of a few isolated outliers.

Table 7 reports one-sided pairwise Wilcoxon signed-rank tests with Holm correction, using the alternative hypothesis that U-HSIC-ENet performs better than each baseline. For NMI, all Holm-corrected p-values are 0.01953, indicating statistically significant improvements over every compared method. For the ARI and ACC, all corrected p-values are 0.03906, again supporting the performance advantage of U-HSIC-ENet after multiple-comparison adjustment. Taken together, Table 5, Table 6 and Table 7 show that the proposed method is supported not only by average scores but also by both global and pairwise statistical evidence. The strongest support appears on NMI, while the ARI and ACC also reach significance under the eight-dataset setting.

**Figure 2:**
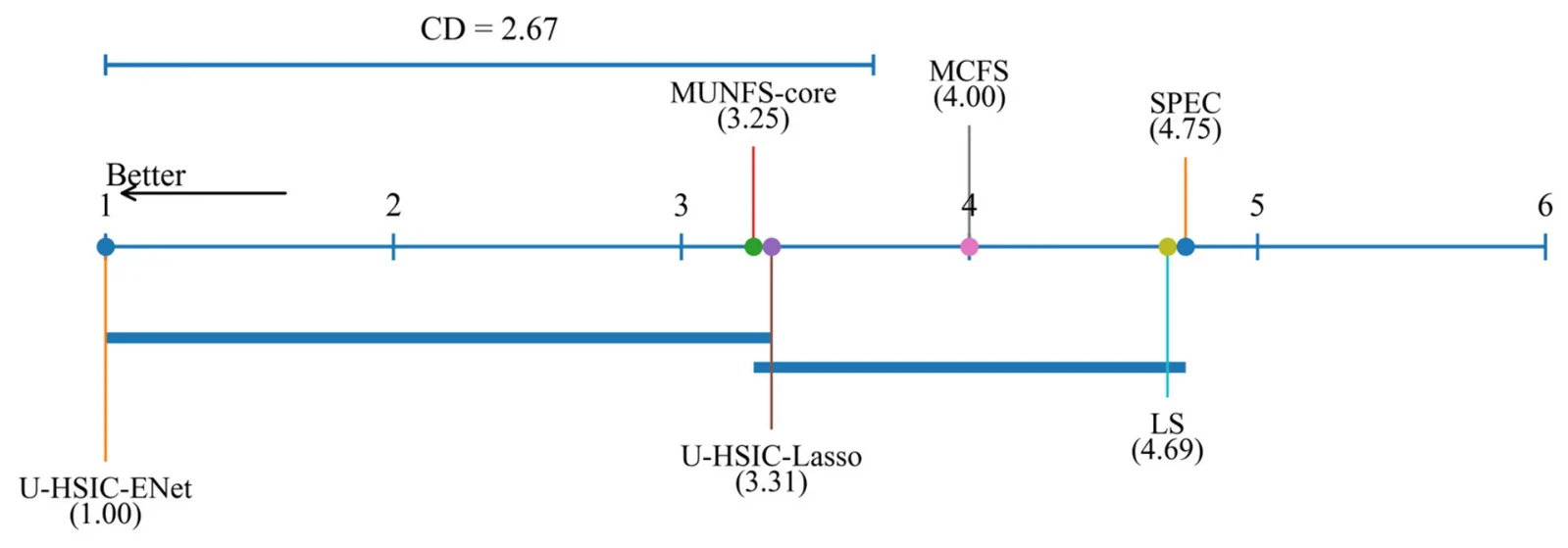
A critical difference (CD) diagram for fixed-k NMI across the eight benchmark datasets. The horizontal axis represents the average rank of each method, with lower ranks indicating better performance. The numbers in parentheses report the corresponding average ranks. The upper horizontal segment indicates the Nemenyi critical difference (CD≈2.67) following the Friedman test, while the thick horizontal bars connect groups of methods whose average rank differences do not exceed the CD. U-HSIC-ENet achieves the best average rank of 1.00. Colors are used only as visual aids to distinguish plotted elements and do not encode additional statistical quantities.

**Figure 3:**
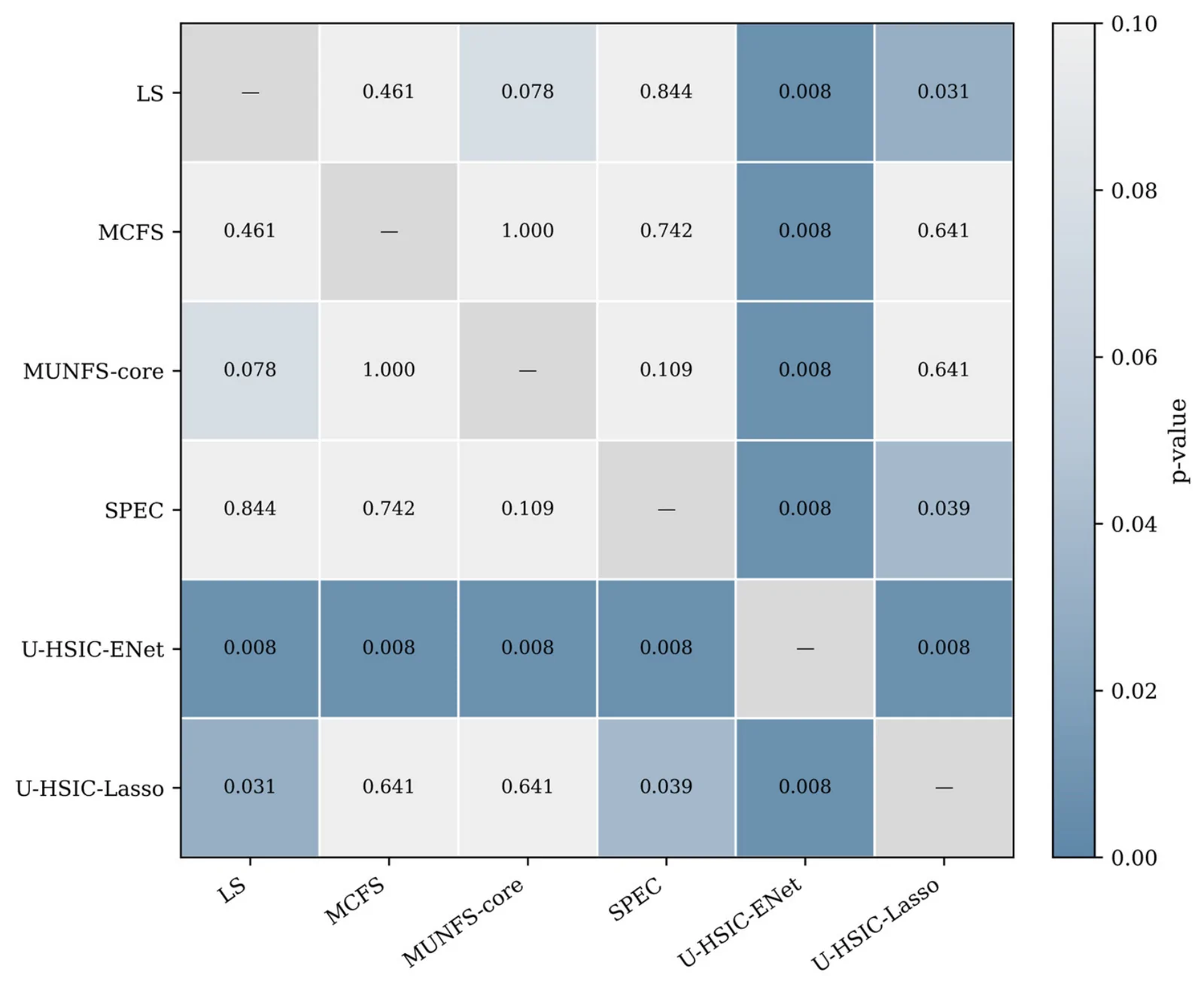
Pairwise Wilcoxon signed-rank *p*-values for fixed-*k* NMI. Smaller *p*-values indicate stronger evidence of performance differences. The heatmap reports unadjusted pairwise *p*-values.

Figure 2 and Figure 3 provide complementary visual summaries of the statistical comparisons for fixed-k NMI. Figure 2 presents the average rank of each method across the eight datasets using the critical difference (CD) framework following the Friedman test [44], while the interpretation of post hoc multiple comparisons follows the framework discussed by García and Herrera [45]. A lower average rank indicates better overall performance. U-HSIC-ENet achieves the lowest possible average rank of 1.00, confirming that it ranks first on all eight datasets. Under the Nemenyi post hoc criterion, two methods are considered significantly different when the difference between their average ranks exceeds the critical difference. With CD≈2.67, the rank advantage of U-HSIC-ENet exceeds the CD relative to MCFS, LS, and SPEC, whereas the differences relative to MUNFS-core and U-HSIC-Lasso do not exceed this threshold. Figure 3 complements this rank-based analysis by displaying the pairwise Wilcoxon signed-rank results. Together with Table 5, Table 6 and Table 7, these analyses provide complementary evidence regarding the overall ranking and pairwise performance differences among the compared methods.

### 4.4. Stability Analysis

To complement the clustering-oriented comparisons, we further evaluate selection stability under data perturbation. Feature selection stability has been regarded as an important complementary criterion because a method may achieve good downstream performance while producing highly variable feature subsets under small data changes [20]. Table 8 reports Jaccard, Kuncheva, and Spearman (Weights) scores obtained under 80% subsampling with 20 repeated subsamples for each dataset. The Jaccard index measures the overlap consistency of selected feature subsets [47]. The Kuncheva index further adjusts selected subset overlap by considering chance agreement under a fixed selection budget [48]. Spearman-based correlation evaluates the consistency of feature rankings or learned weights across subsamples [49].

Table 8 shows that U-HSIC-ENet does not provide uniformly superior selection stability. Its mean Jaccard, Kuncheva, and Spearman scores exceed those of MCFS but are lower than those of the other four compared methods. Thus, the clustering performance improvements reported in Table 2, Table 3 and Table 4 are accompanied by a trade-off in selection reproducibility under subsampling. The ℓ_2_ term serves as a regularization mechanism; its inclusion does not by itself establish superior empirical stability.

These findings distinguish downstream clustering effectiveness from feature selection stability. Although U-HSIC-ENet achieves the highest average clustering scores under the fixed-budget protocol, several baselines produce more reproducible subsets or weight rankings under sample perturbation. Both aspects should therefore be considered when interpreting the empirical performance of the proposed method.

### 4.5. Budget Robustness and Supplementary Visual Analysis

**Figure 4:**
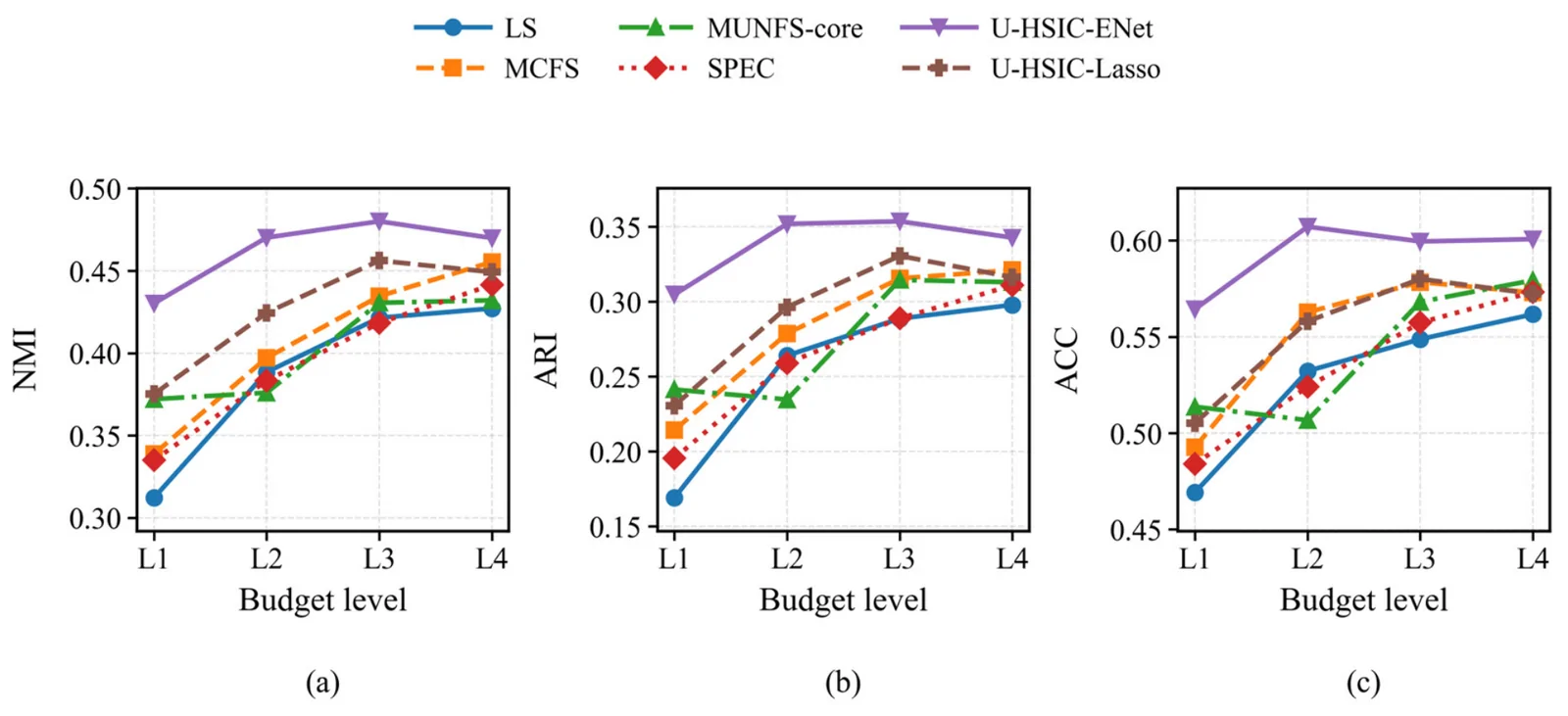
Cross-dataset performance trends across four ordered budget levels. (**a**) NMI, (**b**) the ARI, and (**c**) ACC. For each dataset, L1–L4 denote the evaluated budgets ordered from the smallest to the largest and are used for alignment rather than identical raw *k*/*d* ratios.

The fixed-budget results above establish the main empirical conclusion at the recommended budget $k^{*}$, but a reliable unsupervised feature selection method should also behave reasonably across nearby budget choices. To examine this aspect, Figure 4 summarizes the cross-dataset performance trends in NMI, the ARI, and ACC across four ordered budget levels. Because the eight datasets have different original dimensionalities, the tested budgets cannot be aligned by identical raw k/d ratios. Therefore, the budgets for each dataset are mapped to four ordered levels, denoted by L1–L4 from the smallest to the largest budget, so that the figure reflects relative low-to-high budget behavior rather than exact equality of raw feature proportions.

Figure 4 serves as a supplementary robustness analysis rather than a replacement for the fixed-k comparisons. Its purpose is to show whether the relative advantages observed at $k^{*}$ persist as the feature budget varies from smaller to larger regimes. Combined with the fixed-budget tables and the statistical comparisons above, the trend plots support the conclusion that the good performance of U-HSIC-ENet is not merely an artifact of a single budget choice. Overall, Figure 4 shows that the clustering performance advantage of U-HSIC-ENet persists across the evaluated budget levels. This consistency across feature budgets should be distinguished from feature selection stability under subsampling. As shown in Table 8, several baselines achieve higher selection stability scores, indicating a trade-off between clustering effectiveness and selection reproducibility.

### 4.6. Parameter Sensitivity Analysis

**Figure 5:**
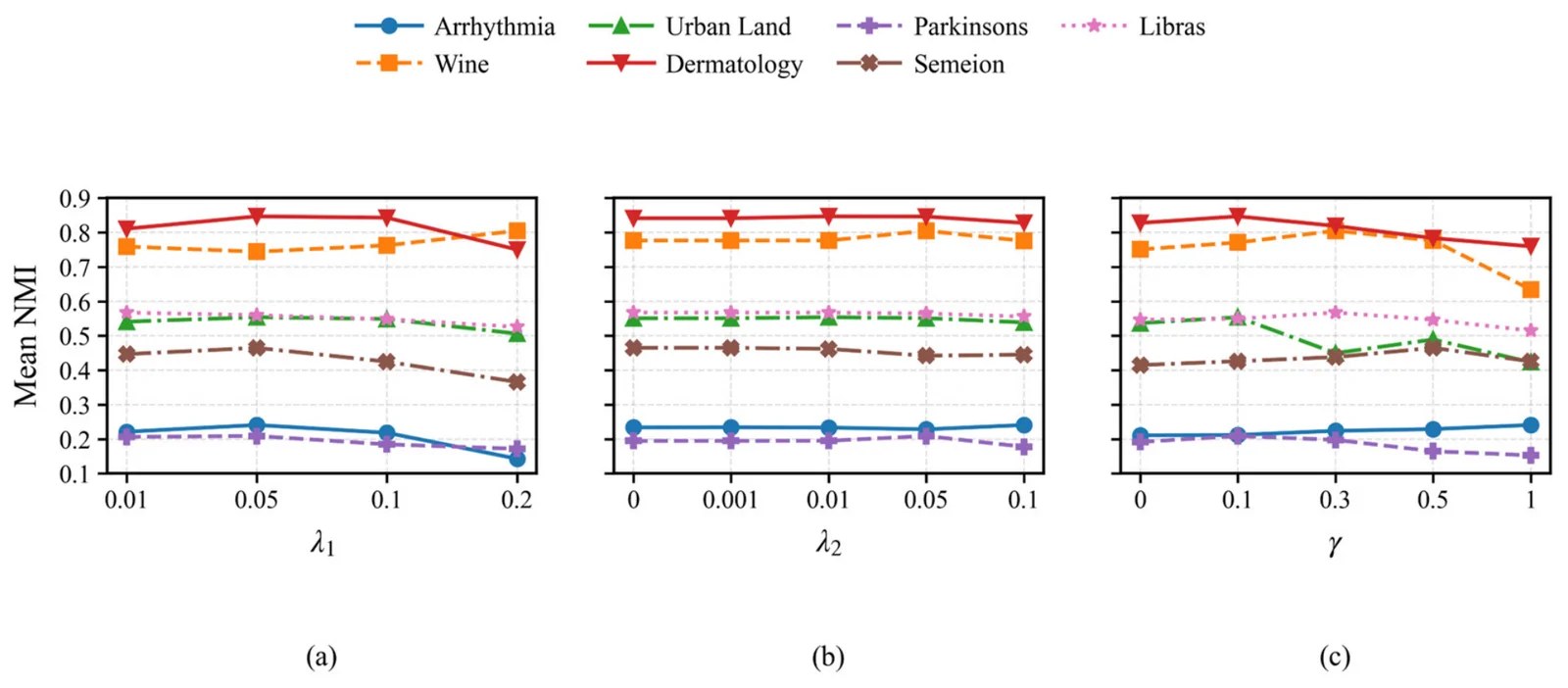
The parameter sensitivity of U-HSIC-ENet. (**a**) Sensitivity to the sparsity parameter $\lambda_{1}$; (**b**) sensitivity to the stabilization parameter $\lambda_{2}$; and (**c**) sensitivity to the redundancy control parameter γ. For each parameter, the remaining two parameters are fixed at their dataset-specific selected values, and each point represents the mean NMI across all evaluated feature budgets and repeated runs. Sonar is omitted from the visualization because its NMI values remain close to zero across the evaluated settings, making the corresponding curve visually uninformative under the common NMI scale.

To investigate the sensitivity of U-HSIC-ENet to its main regularization parameters, we vary $\lambda_{1}$, $\lambda_{2}$, and γ one at a time over their predefined grids while fixing the remaining two parameters at their dataset-specific selected values. The Gaussian kernel bandwidths are not included as independently tuned parameters because they are determined automatically by the median heuristic described in Section 4.1. Consistent with the original parameter selection protocol, the reported value at each parameter setting is the mean NMI across all evaluated feature budgets and repeated runs. Figure 5 summarizes the resulting sensitivity patterns.

Figure 5 shows that the three parameters exhibit different sensitivity characteristics. For $\lambda_{1}$, most datasets remain relatively stable over small-to-moderate values, whereas larger values may cause a noticeable performance decrease on several datasets, indicating that overly strong sparsity can remove informative features. The effect of $\lambda_{2}$ is comparatively mild over most of the evaluated range, with the NMI curves remaining relatively flat on several datasets, which is consistent with its role as a stabilization term. In contrast, γ exhibits stronger dataset dependence. Moderate redundancy penalization is beneficial on several datasets, whereas an excessively large γ can reduce performance, suggesting that overly aggressive redundancy suppression may discard complementary information. Overall, the method is reasonably stable over broad portions of the evaluated parameter grids, while dataset-specific tuning remains useful, particularly for $\lambda_{1}$ and γ.

### 4.7. Ablation Analysis

To further isolate the contributions of the explicit redundancy control and $l_{2}$-stabilization components, we conduct a one-factor-at-a-time ablation analysis using the parameter combinations already evaluated in the original grid search. Importantly, the self-supervised target construction is kept identical in all variants. This is also the same target construction used by the U-HSIC-Lasso baseline; therefore, the comparison with U-HSIC-Lasso already controls for the target-side structural signal. In the ablation analysis, $\lambda_{1}$ is fixed at the dataset-specific value selected for the full model, while $\lambda_{2}$ and γ are removed individually. Four variants are considered: Base, with $\lambda_{2}=0$ and γ=0; $+l_{2}$, with the selected $\lambda_{2}$ but γ=0; +Red, with the selected γ but $\lambda_{2}=0$; and Full, corresponding to the complete U-HSIC-ENet formulation.

Consistent with the original parameter selection protocol, Table 9 reports the mean NMI over all evaluated feature budgets and repeated clustering runs. This aggregation avoids attributing component effects to a single feature budget and permits the direct reuse of the original grid search results.

The ablation results reveal a clear difference in the contributions of the two additional components. Removing both explicit redundancy control and $l_{2}$ stabilization reduces the average NMI from 0.4626 to 0.4338. Introducing $l_{2}$ stabilization alone yields a comparatively modest change, with an average NMI of 0.4351, whereas introducing the explicit redundancy term alone increases the average NMI to 0.4553. The full model achieves the highest average NMI of 0.4626 and is superior to the +Red variant on five datasets while tying on the remaining three. These results indicate that explicit off-diagonal redundancy control accounts for the larger part of the clustering-oriented improvement, whereas the additional contribution of $l_{2}$ stabilization is smaller and dataset-dependent. This observation is consistent with the intended role of the $l_{2}$ term, which primarily provides regularization and numerical stabilization rather than guaranteeing a uniform increase in clustering performance.

### 4.8. Empirical Runtime Analysis

To complement the complexity analysis in Section 3.4, we measured the feature selection runtime of U-HSIC-Lasso and U-HSIC-ENet on Wine, Arrhythmia, and Semeion using the parameter settings reported in Section 4.1. The experiments were conducted on an AMD Ryzen 9 7945HX processor (Advanced Micro Devices, Inc., Santa Clara, CA, USA) under Windows 11 with Python 3.12.7, NumPy 1.26.4, SciPy 1.11.4, and scikit-learn 1.4.2. Numerical library threads were limited to one. Each method was timed independently three times, including construction of the target kernel and feature-wise kernels construction, relevance and interaction matrix computation, optimization, and feature ranking. Data loading, preprocessing, parameter search, and subsequent clustering were excluded. All kernel quantities were rebuilt for each timed run, and the execution order of the two methods was alternated across repetitions and datasets.

Table 10 shows comparable observed runtimes for the two methods on the evaluated datasets. The recorded component timings indicate that kernel and matrix construction dominate the total runtime, particularly on Arrhythmia and Semeion, consistent with the computational burden discussed in Section 3.4. Although U-HSIC-ENet has a lower mean total runtime on Wine and Semeion, these measurements do not establish an algorithmic speed advantage, since both methods share the same kernel construction procedure and its measured duration varies between runs. The reported values characterize execution under the original stopping settings rather than verified time to convergence for every dataset. These results provide practical timing information for the evaluated problem sizes under the stated hardware and implementation settings.

## 5. Conclusions and Future Work

This paper proposed U-HSIC-ENet, an unsupervised feature selection framework based on self-supervised HSIC/CKA and Elastic Net-type regularization. The method addresses the difficulty of identifying informative features without class labels while reducing redundant selections under nonlinear dependencies. By constructing a target kernel directly from unlabeled data, U-HSIC-ENet uses global sample relationships as a proxy structural signal. Feature relevance is measured by the alignment between each feature-induced kernel and the self-supervised target kernel, while redundancy is modeled through pairwise alignment among feature-induced kernels. The main strength of U-HSIC-ENet is its unified treatment of nonlinear relevance, redundancy suppression, sparsity, and stability within a single nonnegative feature weighting objective. The experimental results show that U-HSIC-ENet achieves the highest average clustering scores under the fixed-budget protocol, with particularly clear advantages in NMI. However, several baselines achieve higher selection stability scores under subsampling, indicating that the clustering performance gains are accompanied by a trade-off in selection reproducibility.

The method also has limitations. Constructing feature-wise kernels and the pairwise interaction matrix can be costly when both the sample size and feature dimensionality are large. A sparse HSIC with surrogate kernels provides a possible way to reduce dependence-based selection cost [38], ultra-high-dimensional nonlinear feature selection highlights the importance of scalability [39], and Block HSIC Lasso shows that block-wise computation can support large-scale HSIC-based selection [40]. In addition, U-HSIC-ENet still depends on kernel bandwidths and regularization parameters. More adaptive parameter selection is therefore needed. The current evaluation is mainly clustering-oriented, and broader downstream learning tasks should be examined in future work.

Another limitation concerns self-inclusion in the full-data target kernel. A feature may receive a higher alignment score partly because it contributes to the sample relationships used to construct the target. The present experiments do not separately quantify this effect, so its influence on feature rankings cannot be assumed to be negligible. A leave-one-feature-out alternative would construct the target for each feature using the remaining features, thereby excluding its direct contribution. However, this approach requires additional feature-specific target construction and replaces the common target with a different reference for each feature. It also does not eliminate dependencies between the evaluated feature and correlated remaining features. Evaluating how this alternative affects feature rankings, clustering performance, and computational cost is an important direction for future work.

The proposed framework provides a dependence-based perspective on unsupervised feature selection in which feature relevance and feature redundancy are represented within the same normalized kernel alignment space. Compared with graph-based structural modeling, this formulation offers a complementary approach for characterizing nonlinear relationships without explicitly relying on neighborhood graphs or pseudo-label construction. The resulting representation also makes the distinction between relevance promotion and redundancy suppression more explicit within a single sparse feature weighting framework.

Future work will also focus on scalable kernel approximation, adaptive bandwidth and regularization selection, and alternative nonlinear dependence measures. Recent information-theoretic research has continued to investigate nonparametric dependence measures beyond conventional HSIC-based criteria [50], providing a possible direction for extending the current kernel alignment formulation. Extensions to multiview, streaming, and heterogeneous feature spaces will also be investigated to improve the scalability and adaptability of dependence-based unsupervised feature selection.

## Figures and Tables

**Table 1:** Datasets, fixed budgets, and selected U-HSIC-ENet parameters.

Dataset	*n*	*d*	k*	k*/*d*	$\lambda_{1}$	$\lambda_{2}$	γ
Arrhythmia	452	262	30	0.115	0.05	0.10	1.00
Wine	178	13	2	0.154	0.20	0.05	0.30
Urban Land	675	147	10	0.068	0.05	0.01	0.10
Sonar	208	60	5	0.083	0.10	0.00	1.00
Dermatology	366	34	5	0.147	0.05	0.01	0.10
Parkinsons	195	22	5	0.227	0.05	0.05	0.10
Semeion	1593	256	30	0.117	0.05	0.00	0.50
Libras	360	90	10	0.111	0.01	0.00	0.30

Note: k* denotes the fixed feature budget used for the main comparison and is chosen as the tested budget closest to 0.1d. The reported $\lambda_{1}$, $\lambda_{2}$, and γ are the dataset-specific U-HSIC-ENet parameters selected from the predefined parameter grid using mean NMI across all evaluated feature budgets and repeated runs.

**Table 2:** NMI at fixed budget k*.

Dataset (k*/*d*)	k*	LS	MCFS	MUNFS-Core	SPEC	U-HSIC-Lasso	U-HSIC-ENet
Arrhythmia (0.115)	30	0.1453 ± 0.0062	0.1960 ± 0.0037	0.1471 ± 0.0177	0.1442 ± 0.0061	0.2129 ± 0.0063	**0.2457 ± 0.0132**
Wine (0.154)	2	0.6405 ± 0.0000	0.6342 ± 0.0000	0.5786 ± 0.0000	0.6343 ± 0.0000	0.6405 ± 0.0000	**0.7277 ± 0.0000**
Urban Land (0.068)	10	0.4733 ± 0.0049	0.5080 ± 0.0075	0.5188 ± 0.0127	0.4976 ± 0.0022	0.4940 ± 0.0017	**0.5485 ± 0.0021**
Sonar (0.083)	5	0.0000 ± 0.0000	0.0000 ± 0.0000	0.0002 ± 0.0000	0.0001 ± 0.0000	0.0000 ± 0.0000	**0.0005 ± 0.0000**
Dermatology (0.147)	5	0.5160 ± 0.0000	0.4992 ± 0.0000	0.7061 ± 0.0162	0.6663 ± 0.0115	0.6952 ± 0.0037	**0.7943 ± 0.0047**
Parkinsons (0.227)	5	0.1132 ± 0.0000	0.0628 ± 0.0015	0.2097 ± 0.0059	0.1105 ± 0.0000	0.1644 ± 0.0000	**0.2389 ± 0.0000**
Semeion (0.117)	30	0.4159 ± 0.0104	0.4595 ± 0.0168	0.4162 ± 0.0066	0.4133 ± 0.0055	0.4395 ± 0.0114	**0.5013 ± 0.0054**
Libras (0.111)	10	0.4228 ± 0.0077	0.5812 ± 0.0049	0.5005 ± 0.0115	0.4005 ± 0.0063	0.5156 ± 0.0100	**0.5947 ± 0.0058**
Average		0.3409	0.3676	0.3846	0.3584	0.3953	**0.4564**

Note: Mean ± std over repeated runs; the best result in each row is shown in bold.

**Table 3:** ARI at fixed budget k*.

Dataset (k*/*d*)	k*	LS	MCFS	MUNFS-Core	SPEC	U-HSIC-Lasso	U-HSIC-ENet
Arrhythmia (0.115)	30	0.0309 ± 0.0122	0.0551 ± 0.0058	0.0353 ± 0.0127	0.0171 ± 0.0015	0.0742 ± 0.0120	**0.0812 ± 0.0188**
Wine (0.154)	2	0.5964 ± 0.0000	0.5948 ± 0.0000	0.5491 ± 0.0000	0.6476 ± 0.0000	0.5964 ± 0.0000	**0.7337 ± 0.0000**
Urban Land (0.068)	10	0.3236 ± 0.0056	0.3750 ± 0.0234	0.3526 ± 0.0070	0.3295 ± 0.0036	0.3835 ± 0.0034	**0.4107 ± 0.0147**
Sonar (0.083)	5	−0.0044 ± 0.0000	−0.0044 ± 0.0000	**−0.0033 ± 0.0000**	−0.0047 ± 0.0000	−0.0044 ± 0.0000	−0.0047 ± 0.0000
Dermatology (0.147)	5	0.2119 ± 0.0000	0.2192 ± 0.0000	0.4836 ± 0.0609	0.3897 ± 0.0050	0.4420 ± 0.0004	**0.6394 ± 0.0211**
Parkinsons (0.227)	5	−0.0957 ± 0.0000	0.0480 ± 0.0027	**0.0819 ± 0.0132**	−0.0965 ± 0.0000	−0.0552 ± 0.0000	0.0586 ± 0.0000
Semeion (0.117)	30	0.2599 ± 0.0089	0.3193 ± 0.0262	0.2986 ± 0.0084	0.2452 ± 0.0050	0.3058 ± 0.0104	**0.3847 ± 0.0058**
Libras (0.111)	10	0.1514 ± 0.0132	0.3063 ± 0.0052	0.2264 ± 0.0053	0.1520 ± 0.0047	0.2355 ± 0.0110	**0.3140 ± 0.0103**
Average		0.1842	0.2392	0.2530	0.2100	0.2472	**0.3272**

Note: Mean ± std over repeated runs; the best result in each row is shown in bold.

**Table 4:** ACC at fixed budget k*.

Dataset (k*/*d*)	k*	LS	MCFS	MUNFS-Core	SPEC	U-HSIC-Lasso	U-HSIC-ENet
Arrhythmia (0.115)	30	0.2161 ± 0.0130	0.2552 ± 0.0198	0.2264 ± 0.0122	0.2242 ± 0.0068	0.2861 ± 0.0247	**0.2979 ± 0.0206**
Wine (0.154)	2	0.8371 ± 0.0000	0.8483 ± 0.0000	0.8258 ± 0.0000	0.8764 ± 0.0000	0.8371 ± 0.0000	**0.9045 ± 0.0000**
Urban Land (0.068)	10	0.5481 ± 0.0074	0.5012 ± 0.0337	0.5141 ± 0.0118	0.5412 ± 0.0048	0.5847 ± 0.0023	**0.6030 ± 0.0284**
Sonar (0.083)	5	0.5096 ± 0.0000	0.5096 ± 0.0000	**0.5192 ± 0.0000**	0.5048 ± 0.0000	0.5096 ± 0.0000	0.5048 ± 0.0000
Dermatology (0.147)	5	0.4454 ± 0.0000	0.4508 ± 0.0000	0.6148 ± 0.0315	0.5811 ± 0.0252	0.5665 ± 0.0032	**0.7204 ± 0.0268**
Parkinsons (0.227)	5	0.5692 ± 0.0000	0.6137 ± 0.0030	**0.6513 ± 0.0089**	0.5744 ± 0.0000	0.5231 ± 0.0000	0.6359 ± 0.0000
Semeion (0.117)	30	0.4490 ± 0.0274	0.4878 ± 0.0435	0.4919 ± 0.0170	0.4256 ± 0.0038	0.4873 ± 0.0219	**0.5932 ± 0.0115**
Libras (0.111)	10	0.3204 ± 0.0167	**0.4602 ± 0.0058**	0.3778 ± 0.0048	0.3269 ± 0.0116	0.4037 ± 0.0208	0.4565 ± 0.0397
Average		0.4869	0.5158	0.5277	0.5068	0.5248	**0.5895**

Note: Mean ± std over repeated runs; the best result in each row is shown in bold.

**Table 5:** Friedman test on fixed-*k* results.

Metric	Friedman *χ*^2^	*p*-Value	Datasets	Methods
NMI	22.0863	0.0005	8	6
ARI	16.8345	0.0048	8	6
ACC	12.5912	0.0275	8	6

Note: The Friedman test is conducted across the six comparison methods on the eight datasets.

**Table 6:** Win/tie/loss of U-HSIC-ENet vs. each baseline (fixed k*).

Metric	Baseline	Win	Tie	Loss
NMI	LS	8	0	0
NMI	MCFS	8	0	0
NMI	MUNFS-core	8	0	0
NMI	SPEC	8	0	0
NMI	U-HSIC-Lasso	8	0	0
ARI	LS	7	0	1
ARI	MCFS	7	0	1
ARI	MUNFS-core	6	0	2
ARI	SPEC	7	0	1
ARI	U-HSIC-Lasso	7	0	1
ACC	LS	7	0	1
ACC	MCFS	6	0	2
ACC	MUNFS-core	6	0	2
ACC	SPEC	7	1	0
ACC	U-HSIC-Lasso	7	0	1

Note: Win/tie/loss counts are computed from dataset-level mean performance under the fixed-budget protocol.

**Table 7:** Pairwise Wilcoxon (ours vs. baseline) with Holm correction.

Metric	Baseline	*p*	*p* (Holm)
NMI	LS	0.00391	0.01953
NMI	MCFS	0.00391	0.01953
NMI	MUNFS-core	0.00391	0.01953
NMI	SPEC	0.00391	0.01953
NMI	U-HSIC-Lasso	0.00391	0.01953
ARI	LS	0.00781	0.03906
ARI	MCFS	0.00781	0.03906
ARI	MUNFS-core	0.01953	0.03906
ARI	SPEC	0.00781	0.03906
ARI	U-HSIC-Lasso	0.00781	0.03906
ACC	LS	0.00781	0.03906
ACC	MCFS	0.01953	0.03906
ACC	MUNFS-core	0.01953	0.03906
ACC	SPEC	0.00781	0.03906
ACC	U-HSIC-Lasso	0.00781	0.03906

Note: One-sided Wilcoxon signed-rank tests use the alternative that U-HSIC-ENet performs better than the baseline.

**Table 8:** Stability indicators at fixed k*.

Method	Jaccard	Kuncheva	Spearman (Weights)
MCFS	0.4171 ± 0.1082	0.5057 ± 0.1047	0.6728 ± 0.1712
U-HSIC-ENet	0.5441 ± 0.1712	0.6226 ± 0.1787	0.7075 ± 0.1937
U-HSIC-Lasso	0.5855 ± 0.1574	0.6724 ± 0.1548	0.8453 ± 0.1326
MUNFS-core	0.5909 ± 0.1811	0.6763 ± 0.1581	0.7135 ± 0.1138
LS	0.6920 ± 0.1066	0.7691 ± 0.0956	0.9412 ± 0.0532
SPEC	0.6936 ± 0.1420	0.7693 ± 0.1217	0.9334 ± 0.0392

Note: For each dataset, stability scores were averaged over all 190 pairs of 20 subsamples, each containing 80% of the samples. The table reports the mean and standard deviation of these dataset-level averages across the eight datasets.

**Table 9:** Ablation results in terms of mean NMI across all evaluated feature budgets and repeated runs.

Dataset	Base	$+l_{2}$	+Red	Full
Arrhythmia	0.2120	0.2107	0.2341	**0.2409**
Wine	0.7507	0.7507	0.7766	**0.8048**
Urban Land	0.5388	0.5367	0.5509	**0.5539**
Sonar	0.0019	0.0019	**0.0129**	**0.0129**
Dermatology	0.8276	0.8276	0.8412	**0.8468**
Parkinsons	0.1777	0.1917	0.1947	**0.2090**
Semeion	0.4150	0.4150	**0.4649**	**0.4649**
Libras	0.5466	0.5466	**0.5673**	**0.5673**
**Average**	0.4338	0.4351	0.4553	**0.4626**

Note: Base sets $\lambda_{2}=\gamma =0$; +$l_{2}$ retains the selected $\lambda_{2}$ while setting γ=0; +Red retains the selected γ while setting $\lambda_{2}=0$; Full uses both selected parameters. The self-supervised target kernel and dataset-specific $\lambda_{1}$ are held fixed across the four variants. The best result in each row is shown in bold.

**Table 10:** Feature selection runtime in seconds under the original stopping settings (mean ± standard deviation over three runs).

Dataset	*n*	*d*	U-HSIC-Lasso	U-HSIC-ENet
Arrhythmia	452	262	2.4405 ± 0.2038	2.4745 ± 0.3272
Wine	178	13	0.0136 ± 0.0014	0.0125 ± 0.0003
Semeion	1593	256	34.6278 ± 5.5815	31.3574 ± 0.7318

Note: The fixed feature budgets were 2, 30, and 30 for Wine, Arrhythmia, and Semeion, respectively. The convergence tolerance was $10^{-7}$, and the maximum number of iterations was 800. U-HSIC-ENet reached the 800-iteration limit in all three Semeion runs. The original compact kernel bank implementation was retained, including memory-mapped storage on Semeion and its associated I/O overhead.

## Data Availability

The data that support the findings of this study are available from the corresponding author upon request. There are no restrictions on data availability.

## References

[1] Guyon I., Elisseeff A. (2003). An introduction to variable and feature selection. J. Mach. Learn. Res..

[2] Li J., Cheng K., Wang S., Morstatter F., Trevino R.P., Tang J., Liu H. (2017). Feature selection: A data perspective. ACM Comput. Surv..

[3] Dy J.G., Brodley C.E. (2004). Feature selection for unsupervised learning. J. Mach. Learn. Res..

[4] He X., Cai D., Niyogi P. Laplacian score for feature selection. Proceedings of the 18th International Conference on Neural Information Processing Systems (NIPS 2005).

[5] Zhao Z., Liu H. Spectral feature selection for supervised and unsupervised learning. Proceedings of the 24th International Conference on Machine Learning (ICML 2007).

[6] Cai D., Zhang C., He X. Unsupervised feature selection for multi-cluster data. Proceedings of the 16th ACM SIGKDD International Conference on Knowledge Discovery and Data Mining.

[7] Yan Z., Ma Z., Ma J., Li H. (2025). Robust unsupervised feature selection algorithm based on fuzzy anchor graph. Entropy.

[8] Gretton A., Bousquet O., Smola A., Schölkopf B. Measuring statistical dependence with Hilbert–Schmidt norms. Proceedings of the 16th International Conference on Algorithmic Learning Theory (ALT 2005).

[9] Wang T., Dai X., Liu Y. (2021). Learning with Hilbert–Schmidt independence criterion: A review and new perspectives. Knowl.-Based Syst..

[10] Yamada M., Jitkrittum W., Sigal L., Xing E.P., Sugiyama M. (2014). High-dimensional feature selection by feature-wise kernelized Lasso. Neural Comput..

[11] Wang T., Lu J., Zhang G. (2018). Two-stage fuzzy multiple kernel learning based on Hilbert–Schmidt independence criterion. IEEE Trans. Fuzzy Syst..

[12] Wang T., Lai Z., Zhang X. (2026). Dually feature-weighted fuzzy SVM based on HSIC LASSO. Fuzzy Sets Syst..

[13] Wang T. (2026). A survey on Hilbert–Schmidt independence criterion Lasso. Knowl.-Based Syst..

[14] Battiti R. (1994). Using mutual information for selecting features in supervised neural net learning. IEEE Trans. Neural Netw..

[15] Yu L., Liu H. (2004). Efficient feature selection via analysis of relevance and redundancy. J. Mach. Learn. Res..

[16] Peng H., Long F., Ding C. (2005). Feature selection based on mutual information: Criteria of max-dependency, max-relevance, and min-redundancy. IEEE Trans. Pattern Anal. Mach. Intell..

[17] Brown G., Pocock A., Zhao M.-J., Luján M. (2012). Conditional likelihood maximisation: A unifying framework for information theoretic feature selection. J. Mach. Learn. Res..

[18] Zou H., Hastie T. (2005). Regularization and variable selection via the elastic net. J. R. Stat. Soc. Ser. B Stat. Methodol..

[19] Shah R.D., Samworth R.J. (2013). Variable selection with error control: Another look at stability selection. J. R. Stat. Soc. Ser. B Stat. Methodol..

[20] Nogueira S., Sechidis K., Brown G. (2018). On the stability of feature selection algorithms. J. Mach. Learn. Res..

[21] Cortes C., Mohri M., Rostamizadeh A. (2012). Algorithms for learning kernels based on centered alignment. J. Mach. Learn. Res..

[22] Wang T., Zhao D., Tian S. (2015). An overview of kernel alignment and its applications. Artif. Intell. Rev..

[23] Kornblith S., Norouzi M., Lee H., Hinton G. Similarity of neural network representations revisited. Proceedings of the 36th International Conference on Machine Learning (ICML 2019).

[24] Wang T., Qiu Y., Hua J. (2020). Centered kernel alignment inspired fuzzy support vector machine. Fuzzy Sets Syst..

[25] McInnes L., Healy J., Saul N., Großberger L. (2018). UMAP: Uniform manifold approximation and projection. J. Open Source Softw..

[26] Wang Y., Huang M., Zhou L., Che H., Jiang B. (2024). Multi-cluster nonlinear unsupervised feature selection via joint manifold learning and generalized Lasso. Expert Syst. Appl..

[27] Huang Q., Xia T., Sun H., Yamada M., Chang Y. (2020). Unsupervised nonlinear feature selection from high-dimensional signed networks. Proc. AAAI Conf. Artif. Intell..

[28] Cao C., Zhang Y., Ai Y., Zhang Q. (2026). Unsupervised feature selection via unifying distribution alignment and structure preservation. Inf. Fusion.

[29] Wang T., Hu Z., Liu H. (2023). A unified view of feature selection based on Hilbert–Schmidt independence criterion. Chemom. Intell. Lab. Syst..

[30] Belkin M., Niyogi P., Sindhwani V. (2006). Manifold regularization: A geometric framework for learning from labeled and unlabeled examples. J. Mach. Learn. Res..

[31] Tan J., Gui N., Qiu Z. (2024). GAEFS: Self-supervised graph auto-encoder enhanced feature selection. Knowl.-Based Syst..

[32] Li N., Peng M., Wu Q. (2023). Multiview data clustering with similarity graph learning guided unsupervised feature selection. Entropy.

[33] Zhang B., Suzuki J. (2023). Extending Hilbert–Schmidt independence criterion for testing conditional independence. Entropy.

[34] Fan K., Subedi S., Yang G., Lu X., Ren J., Wu C. (2024). Is Seeing Believing? A Practitioner’s Perspective on High-Dimensional Statistical Inference in Cancer Genomics Studies. Entropy.

[35] Fan K., Subedi S., Dissanayake V., Wu C. (2026). Robust Bayesian high-dimensional variable selection and inference with the horseshoe family of priors. Comput. Stat. Data Anal..

[36] Beck A., Teboulle M. (2009). A fast iterative shrinkage-thresholding algorithm for linear inverse problems. SIAM J. Imaging Sci..

[37] Parikh N., Boyd S. (2014). Proximal algorithms. Found. Trends Optim..

[38] Damodaran B.B., Courty N., Lefèvre S. (2017). Sparse Hilbert Schmidt independence criterion and surrogate-kernel-based feature selection for hyperspectral image classification. IEEE Trans. Geosci. Remote Sens..

[39] Yamada M., Tang J., Lugo-Martinez J., Hodzic E., Shrestha R., Saha A., Ouyang H., Yin D., Mamitsuka H., Sahinalp C. (2018). Ultra high-dimensional nonlinear feature selection for big biological data. IEEE Trans. Knowl. Data Eng..

[40] Climente-González H., Azencott C.-A., Kaski S., Yamada M. (2019). Block HSIC Lasso: Model-free biomarker detection for ultra-high dimensional data. Bioinformatics.

[41] Vinh N.X., Epps J., Bailey J. (2010). Information theoretic measures for clusterings comparison: Variants, properties, normalization and correction for chance. J. Mach. Learn. Res..

[42] Hubert L., Arabie P. (1985). Comparing partitions. J. Classif..

[43] Kuhn H.W. (1955). The Hungarian method for the assignment problem. Nav. Res. Logist. Q..

[44] Demšar J. (2006). Statistical comparisons of classifiers over multiple data sets. J. Mach. Learn. Res..

[45] García S., Herrera F. (2008). An extension on “Statistical comparisons of classifiers over multiple data sets” for all pairwise comparisons. J. Mach. Learn. Res..

[46] Holm S. (1979). A simple sequentially rejective multiple test procedure. Scand. J. Stat..

[47] Jaccard P. (1912). The distribution of the flora in the alpine zone. New Phytol..

[48] Kuncheva L.I. A stability index for feature selection. Proceedings of the IASTED International Conference on Artificial Intelligence and Applications (AIA 2007).

[49] Nogueira S., Sechidis K., Brown G. On the use of Spearman’s rho to measure the stability of feature rankings. Proceedings of the 8th Iberian Conference on Pattern Recognition and Image Analysis (IbPRIA 2017).

[50] Daniušis P., Juneja S., Kuzma L., Marcinkevičius V. (2025). Measuring statistical dependence via characteristic function IPM. Entropy.

